# Distinct OA:PA Ratios Generate Discrete Hepatocellular Phenotypes in HepG2 Cells

**DOI:** 10.3390/cells15191774

**Published:** 2026-09-29

**Authors:** Hangyeon Jeong, Yesol Han, Jiyeong Park, Hanbyul Noh, Eunyoung Moon, Yanghoon Huh

**Affiliations:** Center for Bio-Imaging & Translational Research, Korea Basic Science Institute, Cheongju 28119, Republic of Korea; hangyeon2@kbsi.re.kr (H.J.); yshan01@kbsi.re.kr (Y.H.); jypark37@kbsi.re.kr (J.P.); hbnoh7908@gmail.com (H.N.); mey0916@kbsi.re.kr (E.M.)

**Keywords:** HepG2 cells, oleic acid, palmitic acid, fatty acid ratio, steatosis, lipotoxic stress, hepatocellular phenotype, lipid droplet

## Abstract

**Highlights:**

**What are the main findings?**

- Distinct OA:PA ratios generate discrete hepatocellular states rather than a linear steatosis-to-lipotoxicity continuum
- O2P1 (2:1 OA:PA) co-features lipid accumulation, elevated intracellular ROS, AMPK-associated metabolic stress, NF-κB-associated inflammatory signaling, and measurable IL-8 secretion—selected molecular features associated with MASH-relevant cellular stress.

**What are the implications of the main findings?**

- OA:PA ratio selection in hepatocyte-based in vitro models should be guided by the specific phenotypic dimension under investigation rather than by convention.
- O3P2 may serve as a representative condition for steatosis/LD biology studies; O2P1 for investigations of MASH-relevant stress-responsive signaling; PA for lipotoxicity-focused studies; and O1P2 for PA-enriched inflammatory/ER stress-associated studies.

**Abstract:**

Metabolic dysfunction-associated steatohepatitis (MASH) is characterized by hepatic steatosis, metabolic stress, and inflammation. Although free fatty acid (FFA)-based in vitro models are widely used, how OA:PA ratios shape hepatocellular phenotypes remains incompletely characterized. HepG2 cells were treated with oleic acid (OA), palmitic acid (PA), or four OA:PA mixtures (O1P1, O2P1, O3P2, O1P2) for 24 or 48 h. OA and OA:PA mixtures were evaluated at 500 μM total FFA (O2P1: 333.3 μM OA + 166.7 μM PA; O3P2: 300 μM OA + 200 μM PA), whereas PA alone was evaluated at 200 μM. Cell viability, lipid accumulation, ultrastructure, oxidative response, secreted cytokines, and molecular responses were evaluated. Distinct OA:PA ratios generated discrete hepatocellular states rather than a linear steatosis-to-lipotoxicity continuum. O3P2 produced a steatosis-dominant phenotype with pronounced lipid accumulation and relatively limited stress signaling. In contrast, O2P1 combined substantial lipid accumulation with elevated DCF fluorescence, AMP-activated protein kinase (AMPK) phosphorylation, nuclear factor kappa B (NF-κB)-associated inflammatory signaling, and interleukin-8 (IL-8) secretion, representing a pronounced stress-responsive phenotype with selected MASH-relevant features. Selected responses were also evaluated in SNU-449 cells, revealing cell-line-specific patterns. Collectively, these findings support a phenotype-oriented framework for OA:PA ratio selection in hepatocyte-based in vitro MASH modeling.

## 1. Introduction

Metabolic dysfunction-associated steatotic liver disease (MASLD), including its progressive form metabolic dysfunction-associated steatohepatitis (MASH), has become one of the most prevalent chronic liver diseases worldwide [1,2]. Hepatic steatosis, characterized by excessive accumulation of intracellular lipid droplets (LDs), represents an early pathological stage and is accompanied by metabolic imbalance, inflammatory signaling, organelle dysfunction, and eventual progression toward liver injury and fibrosis [2,3]. Although in vivo models remain essential for investigating disease progression, in vitro hepatic steatosis models provide a convenient and reproducible platform for mechanistic studies and preliminary screening of therapeutic candidates.

Among various in vitro approaches, free fatty acid (FFA)-induced lipid overload models using HepG2 cells are widely employed because of their simplicity and reproducibility [4]. Oleic acid (OA; C18:1, n-9) and palmitic acid (PA; C16:0) are the two most commonly used FFAs for inducing steatotic phenotypes. OA is a monounsaturated fatty acid that preferentially promotes triglyceride synthesis and LD formation with relatively low cytotoxicity [5,6], whereas PA is a saturated fatty acid associated with lipotoxicity, endoplasmic reticulum (ER) stress, mitochondrial dysfunction, and inflammatory responses [7,8,9]. Recent studies and reviews continue to support greater cytotoxicity of PA than OA at equivalent or comparable exposure conditions, supporting the use of lower PA concentrations to maintain experimental viability [4,7,8].

Despite the widespread use of OA/PA-induced steatosis models, substantial variability remains in OA:PA ratios across studies, and the rationale for ratio selection is often limited [10,11]. Early systematic work by Gómez-Lechón et al. [11] compared multiple OA:PA compositions and identified the 2:1 ratio as a condition that induces substantial lipid accumulation with relatively limited cytotoxicity, after which this formulation became widely adopted as a conventional steatosis model. Hetherington et al. [12] further demonstrated that OA, PA, and a combined OA:PA condition elicit distinct lipid-handling and lipotoxic stress responses in HepG2 cells, further supporting fatty acid composition as an important determinant of cellular phenotype. However, direct head-to-head comparisons of alternative OA:PA ratios under matched total FFA exposure remain limited, despite accumulating evidence that OA and PA produce markedly different lipid-storage and stress-associated cellular responses [10]. We therefore hypothesized that OA:PA composition itself represents an experimental variable that determines hepatocellular phenotype rather than merely the magnitude of FFA exposure. Importantly, OA:PA composition may influence not only the extent of intracellular lipid accumulation but also LD morphology, nutrient-sensing responses, and inflammatory signaling, collectively shaping the hepatocellular phenotype generated under a given condition. However, how systematic variation in OA:PA ratio influences integrated hepatocellular responses remains incompletely characterized.

Previous studies have provided important insights into OA/PA-mediated steatotic responses but remain limited in scope. Eynaudi et al. [13] characterized OA/PA effects on LD–mitochondria interactions using a single OA + PA mixture. Gomez-Lechon et al. [11] evaluated FFA-induced steatosis in primary human hepatocytes and HepG2 cells and included TEM-based morphological observations, but did not quantify LD parameters or simultaneously evaluate associated molecular pathways. To our knowledge, few studies have combined quantitative TEM-based LD morphometrics with concurrent assessment of lipogenic, nutrient-sensing, inflammatory, and autophagy/ER stress-related pathways across a systematic series of OA:PA ratios within a unified experimental framework.

Multiple intracellular pathways contribute to the cellular response to FFA overload. Lipogenic regulators including sterol regulatory element-binding protein 1 (SREBP-1) and fatty acid synthase (FASN) contribute to de novo lipogenesis [14]. Energy-responsive signaling pathways involving AMP-activated protein kinase (AMPK) and mechanistic target of rapamycin (mTOR) participate in metabolic adaptation [15], whereas forkhead box O3a (FoxO3a) has been implicated in autophagy- and stress-related regulation [16]. Inflammatory signaling centered on nuclear factor kappa B (NF-κB) contributes to inflammatory gene regulation, including cyclooxygenase-2 (COX-2) and tumor necrosis factor alpha (TNF-α) [17], as well as the chemokine interleukin-8 (IL-8), an NF-κB-responsive mediator of neutrophil recruitment that is secreted extracellularly and has been implicated in hepatic inflammatory responses during MASLD/MASH [18]. In addition, autophagy-related markers [microtubule-associated protein 1 light chain 3 A/B (LC3A/B) and sequestosome 1 (p62/SQSTM1)] and ER stress-associated markers [binding immunoglobulin protein (BiP) and C/EBP homologous protein (CHOP)] are frequently evaluated in studies of lipid overload and hepatocellular stress [19,20].

**Figure 1:**
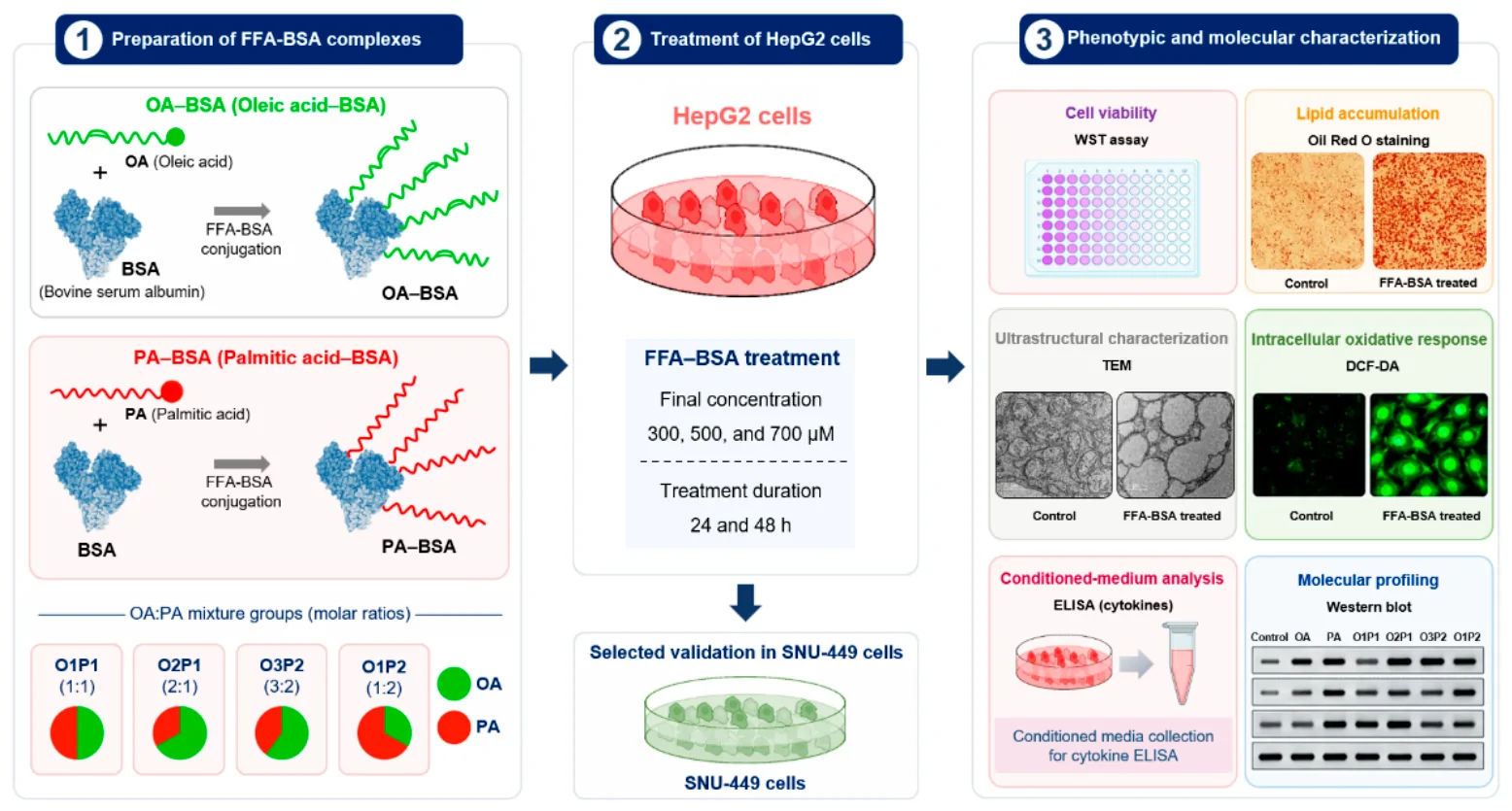
Experimental design and analytical workflow for OA:PA ratio-dependent FFA loading in hepatic cell models. Oleic acid (OA) and palmitic acid (PA) were individually conjugated to fatty acid-free bovine serum albumin (BSA) to generate OA–BSA and PA–BSA complexes. The two FFA–BSA complexes were combined at defined molar ratios to generate O1P1 (OA:PA = 1:1), O2P1 (2:1), O3P2 (3:2), and O1P2 (1:2) treatment conditions. HepG2 cells were exposed to OA and OA:PA mixtures at 300, 500, and 700 μM total FFA, whereas PA was evaluated over a lower concentration range because of its greater cytotoxicity, for 24 or 48 h. Cellular responses were characterized by WST-based cell viability analysis, Oil Red O staining for lipid accumulation, transmission electron microscopy (TEM) for ultrastructural changes, DCF-DA fluorescence for intracellular oxidative responses, cytokine ELISA using conditioned media, and Western blot analysis for molecular profiling. Selected OA:PA-associated responses were additionally evaluated in SNU-449 cells as a secondary cell-line evaluation using representative treatment conditions. OA, oleic acid; PA, palmitic acid; BSA, bovine serum albumin; FFA, free fatty acid; ORO, Oil Red O; TEM, transmission electron microscopy; DCF-DA, 2′,7′-dichlorodihydrofluorescein diacetate; ELISA, enzyme-linked immunosorbent assay. Green and red indicate OA- and PA-derived components, respectively, and arrows indicate the sequential experimental workflow.

In the present study, we systematically compared six FFA conditions—OA alone, PA alone, and four OA:PA mixtures (O1P1, O2P1, O3P2, and O1P2)—in HepG2 cells across multiple concentrations and two exposure durations (Figure 1). The four mixture ratios (1:1, 2:1, 3:2, and 1:2) were selected to span balanced, OA-enriched, intermediate OA-enriched, and PA-enriched compositions under matched total FFA and BSA conditions, rather than to reproduce a single presumed physiological OA:PA ratio. Rather than seeking a single optimal condition, this study aimed to demonstrate that different OA:PA ratios generate discrete hepatocellular states and to establish a phenotype-oriented reference framework for selecting OA:PA conditions according to intended experimental objectives. We further explored whether stress-associated responses scale proportionally with OA:PA composition or instead represent distinct hepatocellular adaptation states.

## 2. Materials and Methods

### 2.1. Preparation of OA/PA Mixtures and FFA–BSA Complexes

Free fatty acid (FFA)–BSA complexes were prepared according to a modified conjugation method. Briefly, a 10% (*w*/*v*) BSA solution was prepared by dissolving 12 g of fatty acid-free bovine serum albumin (BSA; SERVA Electrophoresis, Heidelberg, Germany) in 120 mL of serum-free high-glucose DMEM pre-warmed to 55 °C. Palmitic acid (PA; Sigma-Aldrich, St. Louis, MO, USA, Cat. No. P0500; 256.4 mg) and oleic acid (OA; Sigma-Aldrich, Cat. No. O1008; 282.5 mg) were dissolved in 10 mL of 0.1 N NaOH to achieve 100 mM stock concentrations; PA was heated to 70 °C to ensure complete dissolution. To generate 5 mM FFA–BSA complexes, 1 mL of each 100 mM FFA stock was conjugated with 19 mL of the 10% BSA solution at 55 °C for 10 min, yielding a 5 mM FFA in 9.5% (*w*/*v*) BSA stock with an approximate FFA:BSA molar ratio of 3.5:1. For mixed OA:PA conditions, individual FFA–BSA stocks were combined at the indicated volumetric ratios to achieve O1P1, O2P1, O3P2, or O1P2 composition at a constant total FFA concentration. For all treatment groups, the combined volume of OA–BSA, PA–BSA, and vehicle-BSA solutions was maintained at 20% (*v*/*v*) of the final culture volume (20 μL per 100 μL well), resulting in a constant final BSA concentration of 19 mg/mL [1.9% (*w*/*v*)] across all groups. FFA-free vehicle-BSA was prepared identically by combining 19 mL of the 10% (*w*/*v*) BSA solution with 1 mL of 0.1 N NaOH in place of the FFA stock, yielding a vehicle solution matched in BSA and NaOH content to the FFA–BSA stocks. All preparations were sterile-filtered (0.22 µm) and stored at −20 °C until use.

### 2.2. Cell Culture and FFA Treatment

HepG2 cells (ATCC, Manassas, VA, USA) were maintained in DMEM (HyClone, Logan, UT, USA) supplemented with 10% FBS (HyClone), 100 U/mL penicillin, and 100 µg/mL streptomycin (Invitrogen, Carlsbad, CA, USA) at 37 °C in a humidified 5% CO_2_ atmosphere. To induce FFA overloading, HepG2 cells at 70% confluence were exposed to OA, PA, or OA:PA mixtures at the indicated concentrations for 24 or 48 h. For 48 h exposure groups, the FFA–BSA-containing medium was renewed at 24 h with fresh FFA–BSA treatment medium to maintain consistent FFA availability throughout the exposure period. The total FFA concentration was maintained constant across ratio conditions so that observed differences reflect OA:PA composition rather than total lipid dose. Because PA exerts substantially greater cytotoxicity than OA at equivalent concentrations, as reported previously [7,8,9] and confirmed in our data, PA was tested at 50–500 µM compared with OA at 100–1000 µM.

SNU-449 cells (Korean Cell Line Bank, Seoul, Republic of Korea) were maintained in RPMI 1640 medium (HyClone) supplemented with 10% FBS and 1% penicillin/streptomycin at 37 °C in a humidified 5% CO_2_ atmosphere. For secondary cell-line evaluation, SNU-449 cells at 70% confluence were exposed to OA (500 μM), PA (200 μM), O2P1 (500 μM total FFA), or O3P2 (500 μM total FFA) for 24 h under the same FFA–BSA preparation conditions described above. The compositions and component concentrations of the representative FFA treatment conditions used for downstream analyses are summarized in Table 1.

### 2.3. Cell Viability Assay

Cell viability was assessed using the WST (water-soluble tetrazolium) assay (EZ-Cytox; DoGenBio, Seoul, Republic of Korea). Cells were seeded at 1 × 10^4^ cells/well in 96-well plates and treated with OA (100–1000 µM), PA (50–500 µM), or mixed OA:PA ratios at 300, 500, and 700 µM total FFA for 24 or 48 h. EZ-Cytox reagent (10 µL/well) was added directly to each well and incubated for 1–2 h at 37 °C. Formazan absorbance was measured at 450 nm (Tecan, Männedorf, Switzerland). Cell viability was expressed as a percentage relative to the BSA control group.

### 2.4. Oil Red O Staining

Lipid droplet formation was assessed by Oil Red O (ORO) staining. Cells (1 × 10^4^ cells/well) were fixed with 4% formaldehyde (Biosesang, Yongin, Republic of Korea) for 30 min, rinsed with 60% isopropanol (Ducksan, Ansan, Republic of Korea), and stained with ORO solution (Sigma-Aldrich, 0.3% in 60% propylene glycol) for 15 min. Lipid droplets were imaged using an Olympus IX73 light microscope (Olympus, Center Valley, PA, USA). Oil Red O staining was quantified using ImageJ version 1.54g [National Institutes of Health (NIH), Bethesda, MD, USA]. ORO values were normalized to the corresponding WST-derived cell viability values to minimize cell number bias.

### 2.5. Protein Extraction and Western Blot

Cells were lysed in radioimmunoprecipitation assay (RIPA) lysis buffer (Thermo Fisher Scientific, Waltham, MA, USA) containing protease and phosphatase inhibitors. Protein concentrations were determined by bicinchoninic acid (BCA) assay (Thermo Fisher Scientific). Proteins were separated by sodium dodecyl sulfate-polyacrylamide gel electrophoresis (SDS-PAGE), transferred to polyvinylidene difluoride (PVDF) membranes (Bio-Rad, Hercules, CA, USA), blocked with 5% skim milk for 1 h, and incubated with primary antibodies overnight at 4 °C. Primary antibodies (all 1: 1000): AMPKα [Cell Signaling Technology (CST, Danvers, MA, USA) #2532], BAX (CST, Cat. No. 5023), BCL-2 (CST, Cat. No. 15071), BiP (CST, Cat. No. 3177S), CHOP (CST, Cat. No. 2895S), COX-2 (Abcam, Cambridge, UK, Cat. No. ab283574), FASN (CST, Cat. No. 3180S), FoxO3a (CST, Cat. No. 2497S), IL-1β (CST, Cat. No. 12242S), LC3A/B (CST, Cat. No. 4108S), mTOR (CST, Cat. No. 2983), NF-κB p65 (Abcam, Cat. No. ab32536), p62/SQSTM1 (CST, Cat. No. 5114S), phospho-AMPKα Thr172 (CST, Cat. No. 2535), phospho-FoxO3a Ser253 (CST, Cat. No. 9466S), phospho-mTOR Ser2448 (CST, Cat. No. 2971), phospho-NF-κB p65 Ser536 (CST, Cat. No. 3033S), SREBP-1 (CST, Cat. No. 95879S), and TNF-α (CST, Cat. No. 3707S). GAPDH–horseradish peroxidase (HRP) (ServiceBio, Wuhan, China, Cat. No. ZB15004-HRP; 1:5000) served as loading control for the main figure panels; recombinant anti-β-actin–HRP (ServiceBio, Cat. No. ZB15001-HRP; 1:5000) served as loading control for the BAX/BCL-2 blots (Supplementary Figure S1). Anti-mouse (Abcam, Cat. No. ab205719) or anti-rabbit (Abcam, Cat. No. ab205718) HRP-linked secondary antibodies (Abcam; 1:5000) were applied after three Tris-buffered saline with Tween-20 (TBST) washes. Proteins were detected with chemiluminescence reagent (Cytiva, Marlborough, MA, USA) on an Amersham Imager 600 (Cytiva). Band densities were quantified with ImageJ (NIH) and normalized to GAPDH, except BAX and BCL-2, which were normalized to β-actin, and then expressed relative to the BSA control group (set as 1.000).

### 2.6. Transmission Electron Microscopy and Lipid Droplet Quantification

Samples were fixed in 2.5% glutaraldehyde [Electron Microscopy Sciences (EMS), Hatfield, PA, USA; Cat. No. 16220] in 0.1 M cacodylate buffer (pH 7.3), post-fixed with 2% osmium tetroxide (EMS, Cat. No. 19140) and 1.5% potassium ferrocyanide at 4 °C for 1 h, dehydrated through a graded ethanol/propylene oxide series, and embedded in Epon 812 resin (EMS, Cat. No. 14900). Ultrathin sections (~70 nm) were prepared with an EM UC7 ultramicrotome (Leica Microsystems, Wetzlar, Germany), mounted on 150-mesh copper grids, and stained with uranyl acetate (EMS, Cat. No. 22400) and lead citrate (EMS Cat. No. 22410). Sections were examined using the Bio-HVEM System (OC101, JEM-1400 Plus and JEM-1000BEF; JEOL, Tokyo, Japan) at 120 kV at the Korea Basic Science Institute. Lipid droplets are indicated by asterisks (*) in TEM images. For quantitative LD analysis, TEM micrographs (minimum three non-overlapping fields per sample) were analyzed using ImageJ (NIH). LD number per 100 µm2 cytoplasmic area, LD area (% of cytoplasmic area), and mean LD size (µm2) were quantified and averaged across fields.

### 2.7. Intracellular ROS Assessment (DCF-DA Assay)

Intracellular reactive oxygen species (ROS) were measured using 2′,7′-dichlorofluorescin diacetate (DCF-DA; Sigma-Aldrich). A 10 mM DCF-DA stock in DMSO (stored at −20 °C, light-protected) was diluted 1:1000 in PBS to prepare a 10 μM working solution immediately before use. Following FFA–BSA treatment, cells were washed once with PBS and incubated with DCF-DA working solution (100 μL/well in 96-well format) at 37 °C for 30 min, followed by 2–3 PBS washes. Hoechst 33342 (1:10,000; Abcam, Cat. No. ab228551) was applied at 37 °C for 5 min, washed 2–3 times with PBS, and HBSS was added prior to fluorescence image acquisition. DCF-DA total fluorescence area was quantified using ImageJ (NIH) and normalized to Hoechst 33342-stained nuclear area as a surrogate for cell number. Normalized values were expressed as fold of the BSA control. Data represent *n* = 3 independent biological replicates; multiple non-overlapping fields were acquired per replicate and averaged before statistical analysis.

### 2.8. Enzyme-Linked Immunosorbent Assay (ELISA)

Conditioned media were collected from HepG2 cells following 24 h FFA–BSA treatment and centrifuged at 400× g for 5 min to remove cell debris. Secreted IL-8 was quantified using the Human IL-8 ELISA Kit (LabisKoma, Seoul, Republic of Korea; Cat. No. K0331216) and MCP-1 using Human MCP-1 ELISA (LabisKoma, K0331218). IL-6 was assessed using a high-sensitivity kit (LabisKoma, Cat. No. K0331194HS). IL-8 was reliably quantifiable in HepG2 conditioned medium; IL-6 and MCP-1 remained below assay quantification limits. For SNU-449 conditioned media, all three cytokines were quantifiable. Cytokine concentrations were normalized to total cellular protein content determined by BCA assay.

### 2.9. Statistical Analysis

Data are expressed as mean ± standard error of the mean (SEM). Statistical analyses were performed using GraphPad Prism 8 (GraphPad Software, San Diego, CA, USA). For experiments comparing multiple groups across two time points, two-way analysis of variance (ANOVA) with time and treatment as independent factors was applied, followed by Tukey’s multiple comparison test. For single time-point concentration-response experiments, one-way ANOVA with Tukey’s post hoc test was used. Statistical significance was set at *p* < 0.05. All experiments were performed in three independent biological replicates conducted on separate days using independently seeded HepG2 cultures of different passage numbers. For Western blot analyses, each biological replicate provided one independent protein lysate per condition; no within-blot technical replicates were performed. For WST and ORO assays, measurements were obtained from *n* = 6 wells per condition within each biological replicate as technical replicates, and the mean was used as the value for each biological replicate prior to statistical analysis. For imaging-based assays (TEM and DCF-DA), multiple non-overlapping fields were acquired per biological replicate and averaged before statistical analysis; individual fields were not treated as independent biological replicates.

## 3. Results

### 3.1. PA Is Intrinsically More Cytotoxic than OA, Defining Experimentally Sustainable Exposure Conditions

To define experimentally sustainable FFA exposure conditions, HepG2 cells were treated with OA (100–1000 µM) or PA (50–500 µM) for 24 h and 48 h, and cell viability was assessed using the WST assay. The concentration range for PA was intentionally set lower than that for OA, reflecting its greater cytotoxic potency reported in previous studies owing to lipotoxic, ER stress-associated, and pro-apoptotic properties [7,8,9], which was also supported by the present experimental results.

**Figure 2:**
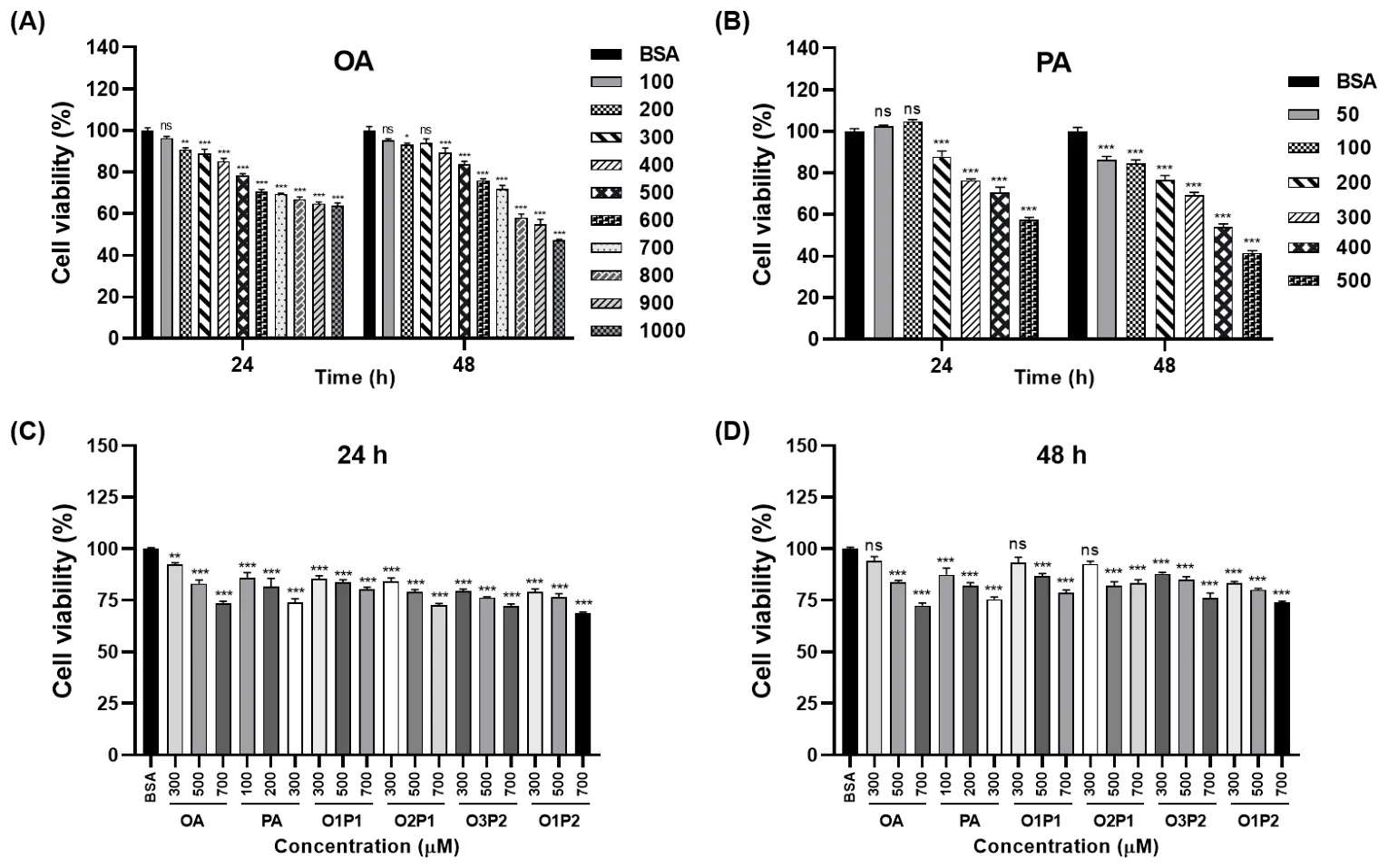
Effects of OA, PA, and different OA:PA ratios on HepG2 cell viability. HepG2 cells were treated with (**A**) OA (100–1000 µM) or (**B**) PA (50–500 µM) for 24 h and 48 h. Cell viability following treatment with mixed OA:PA ratios at 300, 500, and 700 µM for (**C**) 24 h and (**D**) 48 h. OA and OA:PA mixture groups (O1P1, O2P1, O3P2, and O1P2) were evaluated at total FFA concentrations of 300, 500, and 700 μM, whereas PA-alone conditions were evaluated at 100, 200, and 300 μM. Cell viability was determined using the WST assay and expressed relative to the corresponding BSA vehicle control. For 48 h treatments, fresh FFA–BSA-containing medium was provided at 24 h. Data: mean ± SEM (*n* = 3 independent biological replicates; each derived from the mean of 6 technical wells). * *p* < 0.05, ** *p* < 0.01, *** *p* < 0.001 vs. BSA; ns, not significant.

OA reduced viability in a concentration-dependent manner and maintained viability above 70% up to 500 µM at 24 h. Cell viability reached 96.32 ± 0.80%, 88.82 ± 2.17%, 78.36 ± 0.81%, 69.54 ± 0.30%, and 64.85 ± 0.77% at 100, 300, 500, 700, and 900 µM, respectively (Figure 2A). At 48 h, the corresponding values were 95.11 ± 0.87%, 94.15 ± 1.77%, 83.84 ± 1.38%, 71.78 ± 1.74%, and 55.05 ± 2.12%.

In contrast, PA induced marked cytotoxicity at lower concentrations. At 24 h, viability was 102.63 ± 0.36%, 104.85 ± 0.75%, 87.78 ± 2.69%, 76.37 ± 0.64%, and 70.52 ± 2.53% at 50, 100, 200, 300, and 400 µM, respectively (Figure 2B). At 48 h, viability declined to 86.39 ± 1.47%, 84.59 ± 1.72%, 76.62 ± 2.11%, 69.31 ± 1.28%, and 54.11 ± 1.36%, falling below 80% above 200 µM.

These findings indicate that PA and OA exhibit distinct cytotoxic profiles and should not be directly compared at equivalent molar concentrations, as differences in viability may influence downstream phenotypic interpretation. Accordingly, 300, 500, and 700 µM were selected as the working concentrations for OA and OA:PA mixture groups, whereas 200 µM was selected as the representative concentration for PA.

When cells were treated with mixed OA:PA ratios at total FFA concentrations of 300, 500, and 700 µM, all conditions maintained viability above 70% at 24 h (Figure 2C,D). At 500 µM and 24 h, viability was 83.13 ± 1.87% (OA), 83.97 ± 1.12% (O1P1), 79.06 ± 1.11% (O2P1), 76.27 ± 0.39% (O3P2), and 76.45 ± 1.07% (O1P2). These conditions were retained for comparative phenotypic analyses, with subsequent findings interpreted in the context of the observed partial reduction in cell viability.

### 3.2. OA-Rich Conditions Preferentially Promote Lipid Storage Despite Differential Cytotoxicity

**Figure 3:**
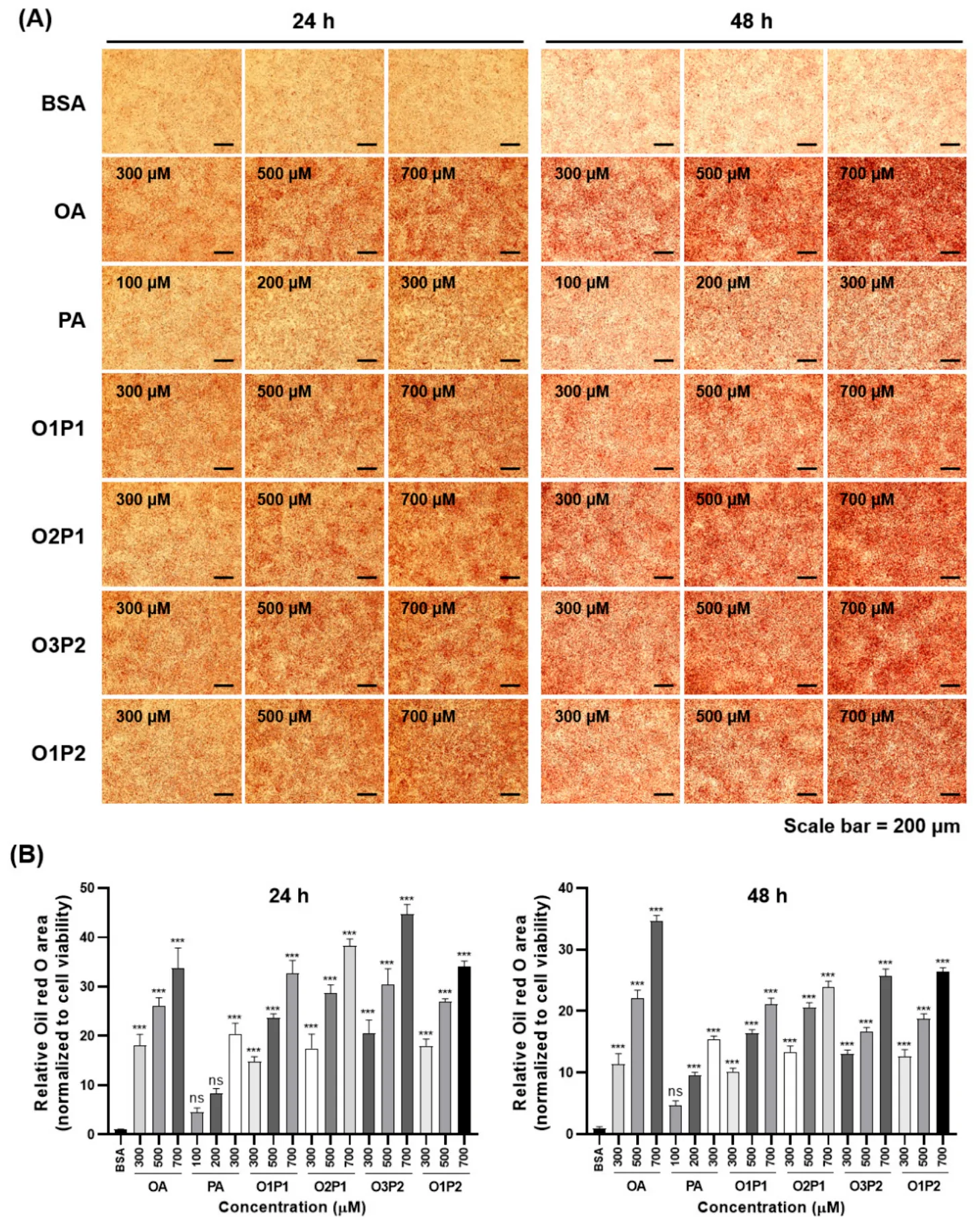
OA:PA composition differentially regulates lipid accumulation in HepG2 cells. (**A**) Representative Oil Red O (ORO) staining images of HepG2 cells treated with OA, PA, or OA:PA mixtures (300, 500, and 700 µM) for 24 h and 48 h. OA and OA:PA mixture groups were treated at total FFA concentrations of 300, 500, and 700 μM, whereas PA-alone groups were treated at 100, 200, and 300 μM. Scale bar = 200 µm. (**B**) Quantification of ORO staining area normalized to WST-derived cell viability (BSA = 1.00). Data are presented as mean ± SEM. Statistical significance was evaluated against the corresponding BSA control. *** *p* < 0.001; ns, not significant.

Intracellular lipid accumulation was assessed by ORO staining normalized to WST-derived cell viability. All FFA-treated groups showed significantly increased ORO signals compared with the BSA control (BSA = 1.00 ± 0.10; *p* < 0.001), with the exception of PA at its representative concentration (200 µM), which did not differ significantly from BSA (ns); PA reached significance at 300 µM (*p* < 0.001; Figure 3). At the representative 24 h conditions (500 µM total FFA for OA and mixtures; 200 µM for PA), normalized ORO staining area values were 26.16 ± 1.59 (OA), 8.36 ± 0.95 (PA), 23.73 ± 0.81 (O1P1), 28.71 ± 1.67 (O2P1), 30.47 ± 3.16 (O3P2), and 26.92 ± 0.65 (O1P2).

Notably, PA produced substantially lower ORO signals than OA despite comparable or greater cytotoxicity, indicating that intracellular lipid accumulation does not directly parallel reductions in cell viability (Figure 3B).

The steatotic phenotype varied according to both OA:PA ratio and total FFA concentration. At 700 µM, O3P2 (44.71 ± 1.96) showed the highest ORO signal and exceeded O2P1 (38.27 ± 1.40) and O1P1 (32.68 ± 2.67). For O1P2, ORO signals increased with concentration at 24 h, reaching 18.03 ± 1.36 (300 µM), 26.93 ± 0.65 (500 µM), and 34.16 ± 1.06 (700 µM) (Figure 3B). These findings indicate that steatotic outcomes were influenced by both fatty acid composition and total FFA exposure rather than by either factor alone.

At 48 h and 500 µM, normalized ORO staining area values were 20.63 ± 0.75 in O2P1 and 16.71 ± 0.65 in O3P2 (Figure 3B).

For subsequent ultrastructural and molecular analyses, 500 μM total FFA was selected as a standardized intermediate dose that produced robust lipid-loading phenotypes while avoiding the greater viability loss observed at higher exposure. PA alone was evaluated at 200 μM because of its greater intrinsic cytotoxicity.

### 3.3. Distinct OA:PA Ratios Generate Structurally Divergent Lipid Droplet Profiles

**Figure 4:**
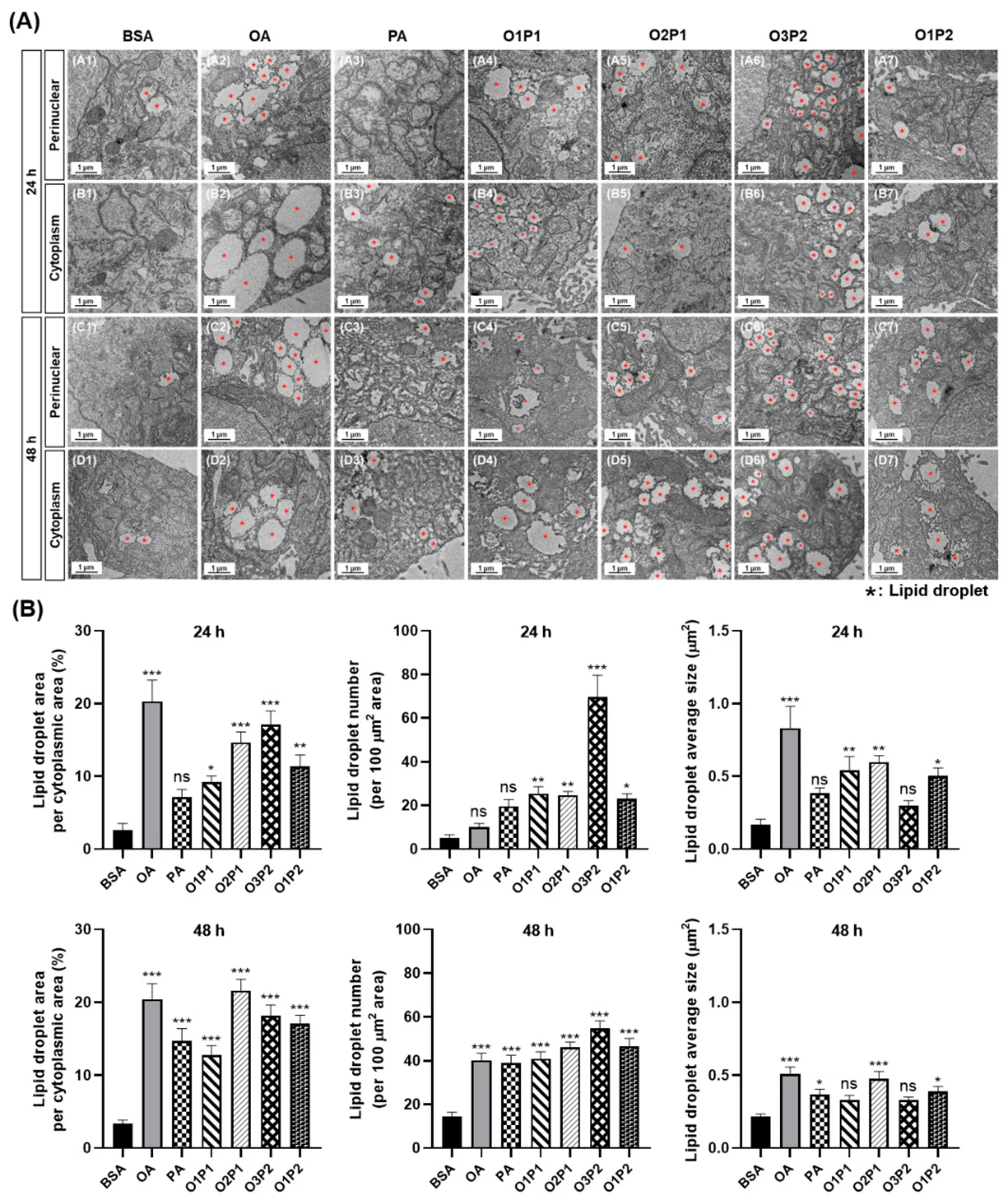
Distinct OA:PA ratios generate structurally divergent lipid droplet profiles. (**A**) Representative TEM images of HepG2 cells treated with BSA, OA (500 µM), PA (200 µM), or OA:PA mixtures (500 µM) for 24 h and 48 h. Perinuclear (rows A, C) and cytoplasmic (rows B, D) regions are shown. Asterisks (*) indicate lipid droplets. Scale bar = 1 µm. (**B**) Quantification of LD area (% cytoplasmic area), LD number (/100 µm^2^), and mean LD size (µm^2^) at 24 h and 48 h. Data are presented as mean ± SEM. Statistical significance was evaluated against the corresponding BSA control. * *p* < 0.05, ** *p* < 0.01, *** *p* < 0.001; ns, not significant. Panels A–D represent the 24 h perinuclear, 24 h cytoplasmic, 48 h perinuclear, and 48 h cytoplasmic regions, respectively. Within each panel, numbers 1–7 correspond to BSA, OA, PA, O1P1, O2P1, O3P2, and O1P2, respectively.

To characterize steatotic phenotypes at the ultrastructural level, TEM was performed on HepG2 cells treated with OA and OA:PA mixtures at 500 µM total FFA and PA alone at 200 µM for 24 h and 48 h. Lipid droplets (LDs) are indicated by asterisks (*) in TEM images (Figure 4A). Quantitative morphometric analyses of LD area, number, and mean size are shown in Figure 4B.

At 24 h, BSA-treated control cells exhibited sparse and small LDs (LD area: 2.65 ± 0.86%; LD number: 5.14 ± 1.41/100 µm^2^; mean size: 0.167 ± 0.039 µm^2^) with preserved intracellular organization. Three representative LD patterns were observed across the treatment conditions.

OA treatment generated large, electron-lucent LDs consistent with neutral lipid-rich storage morphology (mean size: 0.830 ± 0.150 µm^2^; *p* < 0.001 vs. BSA; Figure 4A, panels A2 and B2). In contrast, PA-treated cells displayed smaller and more electron-dense LDs (0.385 ± 0.035 µm^2^), accompanied by mitochondrial swelling and disrupted cristae morphology, representing mitochondrial ultrastructural alterations (Figure 4A, panels A3 and B3).

Among the mixed conditions, O3P2 generated the highest LD number per unit cytoplasmic area at 24 h (69.59 ± 10.05/100 µm^2^; *p* < 0.001), with numerous small-to-moderate LDs (mean size: 0.298 ± 0.036 µm^2^), producing a dispersed microvesicular lipid accumulation pattern (Figure 4A, panels A6 and B6). O2P1 showed an intermediate phenotype (LD area: 14.66 ± 1.44%; LD number: 24.69 ± 1.73/100 µm^2^; mean size: 0.598 ± 0.045 µm^2^), combining substantial lipid storage with moderate ultrastructural stress-associated features (Figure 4A, panels A5 and B5).

At 48 h, substantial LD accumulation remained evident across the examined conditions. LD area reached 20.40 ± 2.13% (OA), 14.77 ± 1.67% (PA), 21.61 ± 1.54% (O2P1), and 18.16 ± 1.45% (O3P2), while LD number reached 40.21 ± 3.25 (OA), 39.04 ± 3.41 (PA), 46.10 ± 2.46 (O2P1), and 54.93 ± 3.37/100 µm^2^ (O3P2).

This persistent structural lipid accumulation was observed even though several molecular markers no longer differed significantly from BSA at 48 h (Figure 4A, rows C and D).

TEM analysis further revealed distinct LD morphologies across FFA conditions. OA-rich conditions were associated with relatively enlarged LD structures, whereas O3P2 was distinguished by a high LD number and relatively smaller mean LD size than OA-rich conditions.

### 3.4. O2P1 Was Associated with Elevated Intracellular ROS at 24 h

**Figure 5:**
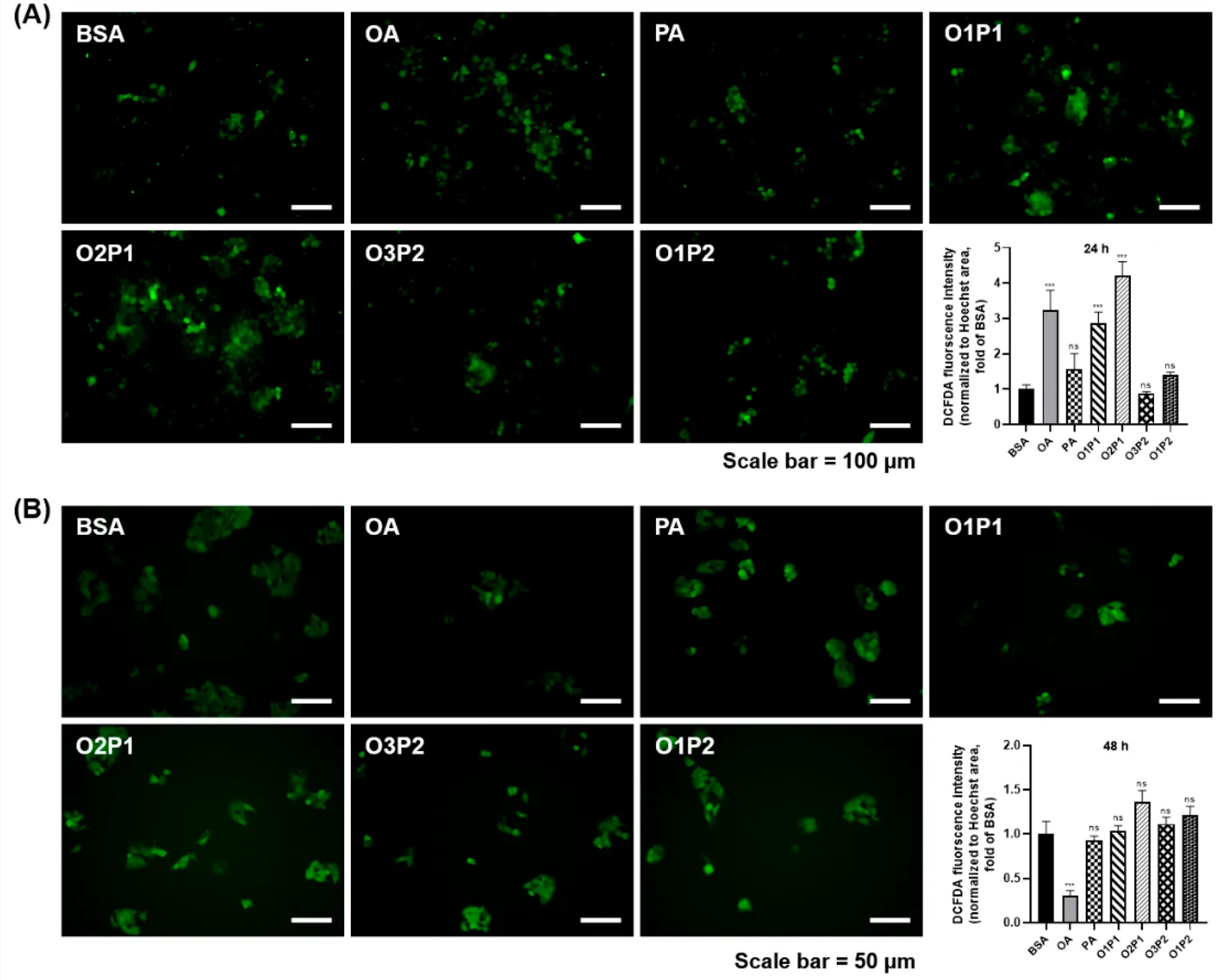
OA:PA composition induces time-dependent intracellular oxidative responses in HepG2 cells. (**A**,**B**) Representative DCF-DA fluorescence images of HepG2 cells treated with BSA vehicle, OA (500 μM), PA (200 μM), O1P1, O2P1, O3P2, or O1P2 (500 μM total FFA) for 24 (**A**) or 48 h (**B**). Intracellular DCF-associated fluorescence was quantified and expressed relative to the corresponding BSA control. For 48 h treatments, fresh FFA–BSA-containing medium was provided at 24 h. Data are presented as mean ± SEM. Statistical significance was evaluated against the corresponding BSA control. *** *p* < 0.001 vs. BSA; ns, not significant. Scale bars are indicated in the images.

Intracellular ROS levels were assessed by DCF-DA fluorescence normalized to Hoechst-stained nuclear area (Figure 5). At 24 h (Figure 5A), O2P1 exhibited the highest DCF-DA fluorescence among all conditions (4.23-fold vs. BSA; *p* < 0.001), followed by OA (3.24-fold; *p* < 0.001) and O1P1 (2.87-fold; *p* < 0.001). PA (1.57-fold), O3P2 (0.87-fold), and O1P2 (1.41-fold) did not differ significantly from BSA, consistent with a comparatively low oxidative-stress profile for O3P2 despite its extensive lipid accumulation. At 48 h (Figure 5B), DCF-DA fluorescence no longer differed significantly from BSA in PA, O1P1, O2P1, O3P2, or O1P2, and was significantly below BSA in OA; this pattern may be consistent with a time-dependent adaptive response, particularly because treatment medium was renewed at 24 h, rather than FFA substrate depletion.

### 3.5. Lipogenic Marker Activation Was Not Proportionally Associated with Intracellular Lipid Accumulation

**Figure 6:**
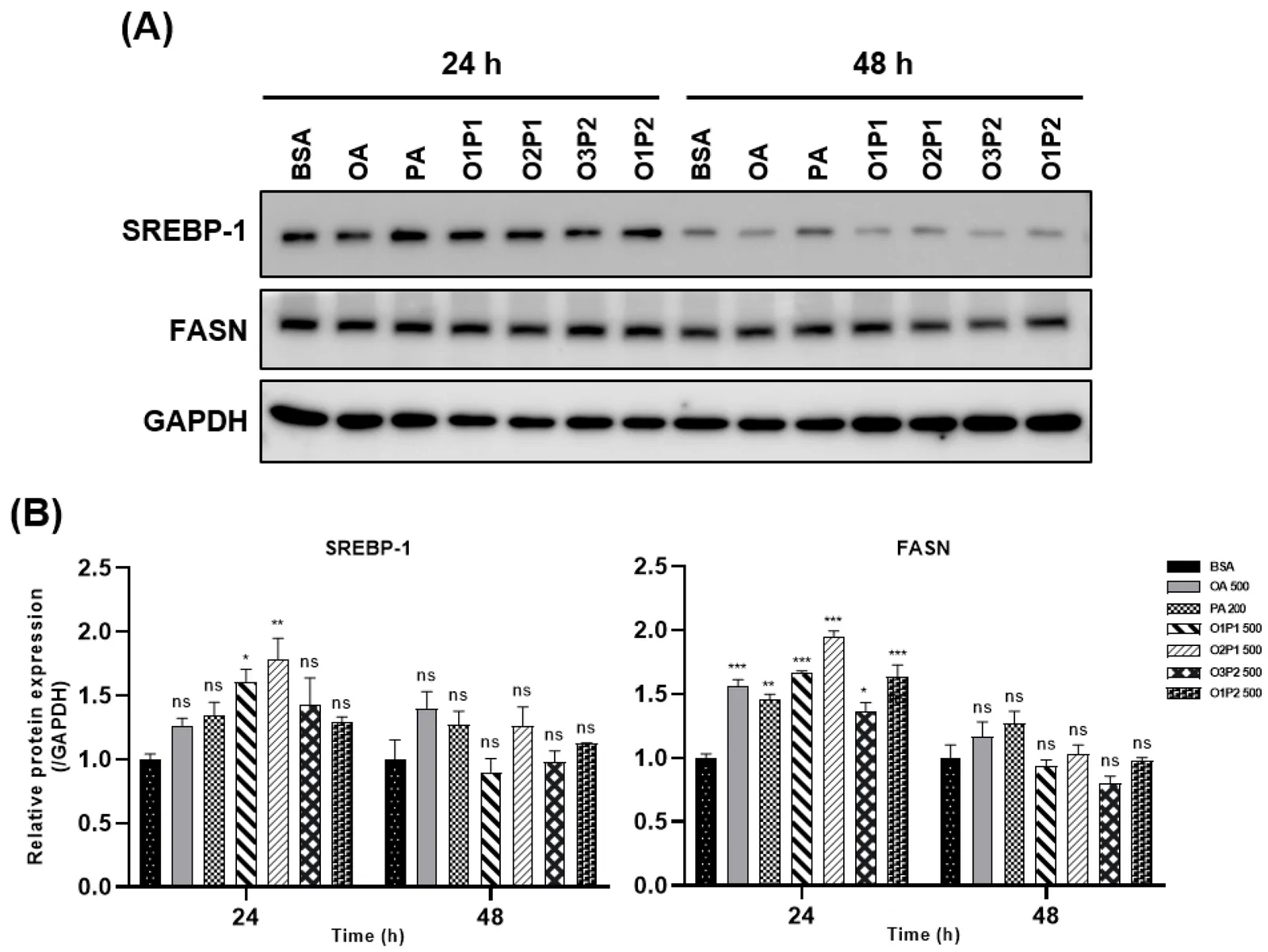
Lipogenic marker activation was not proportionally associated with intracellular lipid accumulation. (**A**) Representative Western blot images of SREBP-1 and FASN in HepG2 cells treated with OA (500 µM), PA (200 µM), or OA:PA mixtures (500 µM) for 24 h and 48 h. GAPDH was used as a loading control. (**B**) Quantification of relative protein expression (normalized to GAPDH; BSA = 1.000). Data: mean ± SEM (*n* = 3). * *p* < 0.05, ** *p* < 0.01, *** *p* < 0.001 vs. BSA; ns, not significant.

To determine whether differential lipid accumulation across OA:PA conditions was associated with lipogenic protein expression, SREBP-1 and FASN expression were analyzed (Figure 6). At 24 h, FASN protein levels were significantly elevated across all FFA conditions, whereas SREBP-1 reached significance only in O1P1 and O2P1. SREBP-1 protein levels were 1.27 ± 0.06 (OA, ns), 1.34 ± 0.10 (PA, ns), 1.61 ± 0.10 (O1P1, *p* < 0.05), 1.79 ± 0.16 (O2P1, *p* < 0.01), 1.43 ± 0.21 (O3P2, ns), and 1.29 ± 0.04 (O1P2, ns) relative to the BSA control (Con = 1.000). FASN levels were 1.56 ± 0.05 (OA, *p* < 0.001), 1.45 ± 0.04 (PA, *p* < 0.01), 1.67 ± 0.01 (O1P1, *p* < 0.001), 1.95 ± 0.04 (O2P1, *p* < 0.001), 1.37 ± 0.07 (O3P2, *p* < 0.05), and 1.64 ± 0.09 (O1P2, *p* < 0.001) (Figure 6A,B).

Notably, lipogenic marker activation did not proportionally parallel intracellular lipid accumulation across conditions. Although O3P2 produced the highest ORO signal at 700 µM, FASN expression at 500 µM remained lower than that observed in O2P1. Likewise, SREBP-1 and FASN induction did not consistently track with LD accumulation across OA:PA ratios.

These findings suggest that differences in lipid accumulation may not be fully explained by changes in lipogenic protein abundance alone and may additionally reflect differences in intracellular lipid handling and storage under the present experimental conditions.

At 48 h, neither SREBP-1 nor FASN levels differed significantly from BSA in any condition, suggesting that the lipogenic protein changes observed at 24 h represent an early, transient response rather than a sustained determinant of phenotypic divergence among OA:PA ratios (Figure 6A,B).

### 3.6. O2P1 Was Associated with Enhanced AMPK Activation Without Major OA:PA Ratio–Dependent Changes in mTOR Phosphorylation

**Figure 7:**
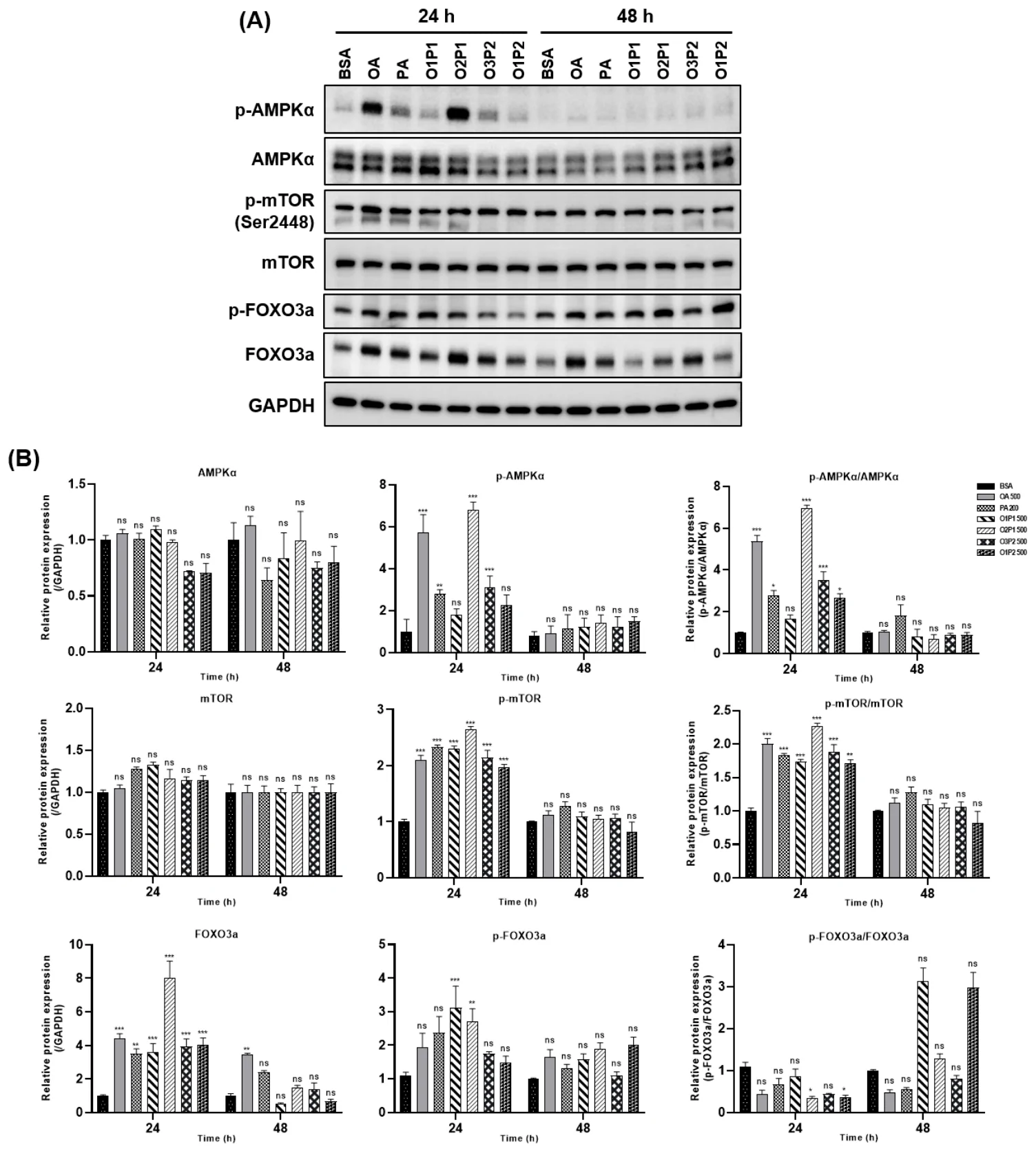
OA:PA composition differentially modulates metabolic stress-responsive signaling in HepG2 cells. (**A**) Representative Western blot images of AMPKα, p-AMPKα, mTOR, p-mTOR (Ser2448), FoxO3a, and p-FoxO3a (Ser253). GAPDH was used as a loading control. (**B**) Quantification of relative protein expression and phosphorylation ratios at 24 h and 48 h. Data: mean ± SEM (*n* = 3). * *p* < 0.05, ** *p* < 0.01, *** *p* < 0.001 vs. BSA; ns, not significant.

Phosphorylation levels of AMPKα, mTOR, and FoxO3a were examined to characterize metabolic stress responses across OA:PA ratio conditions (Figure 7). At 24 h, O2P1 exhibited the highest p-AMPKα/AMPKα ratio (6.97 ± 0.13; *p* < 0.001), followed by OA (5.37 ± 0.29; *p* < 0.001), O3P2 (3.53 ± 0.39; *p* < 0.05), and PA (2.78 ± 0.24; *p* < 0.05), all of which differed significantly from BSA (1.00 ± 0.03); O1P2 (2.65 ± 0.21) and O1P1 (1.67 ± 0.19) did not differ significantly from BSA (Figure 7B). In O2P1, total AMPKα remained close to the BSA level, indicating that the marked increase in the p-AMPKα/AMPKα ratio primarily reflected enhanced AMPK phosphorylation in this condition.

At 48 h, the pronounced O2P1-associated AMPK response was no longer evident (0.68 ± 0.22, not significantly different from BSA). Overall, the strong O2P1-associated AMPK response was therefore most evident at 24 h.

The p-mTOR/mTOR ratio was elevated across most FFA-treated conditions at 24 h, with the highest value observed in O2P1 (2.27 ± 0.04), followed by OA (2.00 ± 0.08) and PA (1.83 ± 0.03). However, unlike p-AMPKα, the pattern of p-mTOR elevation did not exhibit a clear OA:PA ratio-dependent trend, and increases were observed across multiple FFA conditions rather than being uniquely associated with O2P1. These findings suggest that mTOR phosphorylation responded broadly to FFA exposure and contributed less prominently to OA:PA ratio-dependent phenotypic divergence than AMPK signaling. At 48 h, p-mTOR/mTOR ratios did not differ significantly from BSA in any condition (range: 0.83–1.28).

Total FoxO3a abundance increased across all FFA conditions at 24 h, with the greatest increase observed in O2P1 (8.02 ± 1.01), followed by OA (4.40 ± 0.30), O1P2 (4.05 ± 0.41), O3P2 (3.95 ± 0.44), O1P1 (3.62 ± 0.49), and PA (3.50 ± 0.29). Although p-FoxO3a abundance also increased, its increase was smaller relative to that of total FoxO3a, resulting in a p-FoxO3a/FoxO3a ratio that was significantly reduced relative to BSA in OA, O1P1, O2P1, and O3P2 (with the lowest ratio in O2P1) and did not differ significantly from BSA in PA and O1P2 at 24 h (Figure 7B).

At 48 h, FoxO3a responses became more heterogeneous: total and phosphorylated FoxO3a no longer showed the uniform elevation pattern at 24 h, and the p-FoxO3a/FoxO3a ratio was significantly elevated relative to BSA only in O1P1 and O1P2, with the remaining conditions not differing significantly from BSA. Because these late ratio changes were influenced in part by reductions in total FoxO3a abundance in some conditions, the phosphorylation ratio alone should not be interpreted as evidence of enhanced FoxO3a functional activity.

### 3.7. Inflammatory Signaling Diverges by Marker and Condition, Reflecting Multidimensional Responses

**Figure 8:**
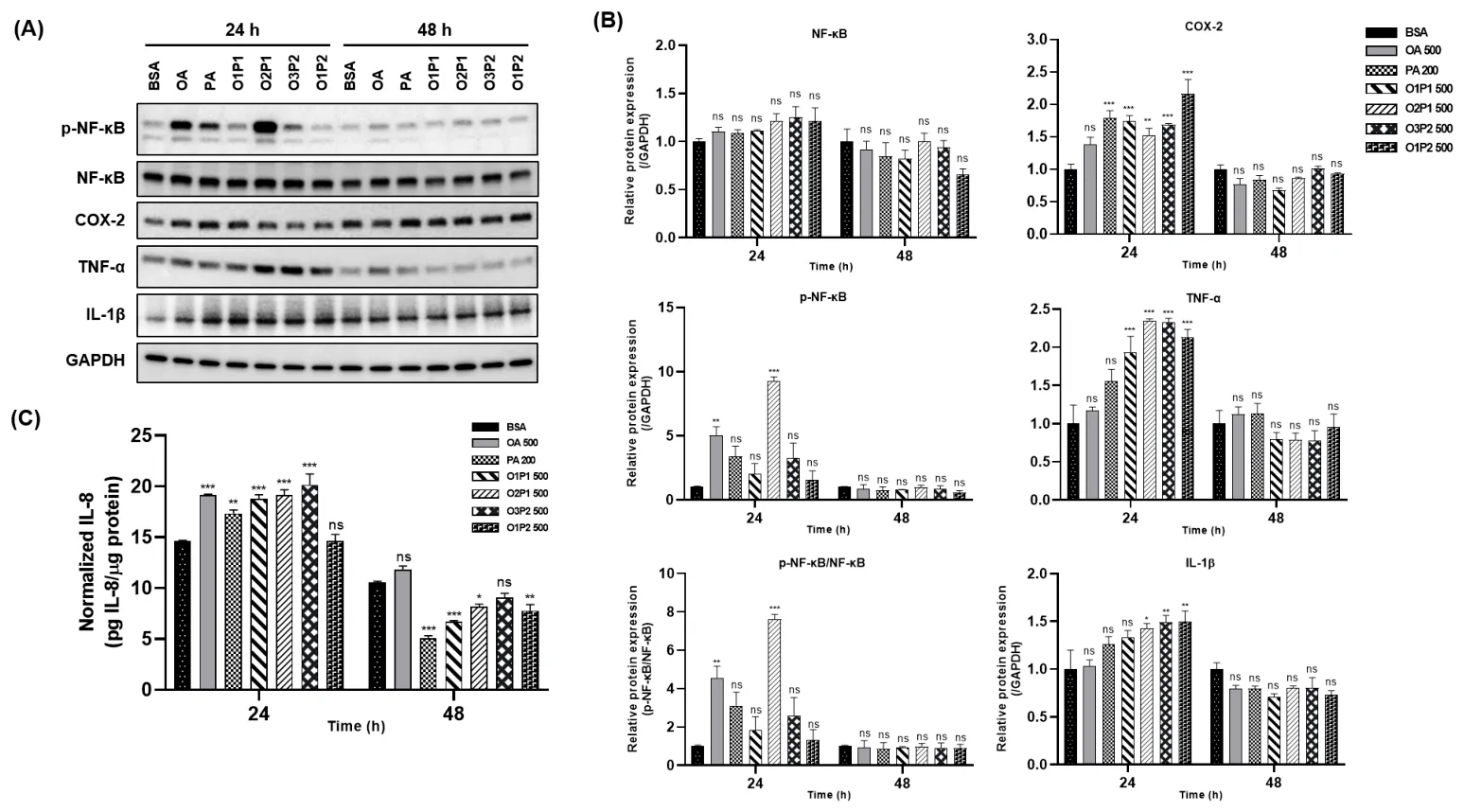
OA:PA composition differentially modulates inflammatory signaling and IL-8 secretion in HepG2 cells. (**A**) Representative Western blot images of p-NF-κB p65 (Ser536), NF-κB p65, COX-2, TNF-α, and IL-1β in HepG2 cells treated with OA (500 µM), PA (200 µM), or OA:PA mixtures (500 µM) for 24 h and 48 h. GAPDH was used as a loading control. (**B**) Quantification of relative protein expression including the p-NF-κB/NF-κB ratio. (**C**) IL-8 concentrations in conditioned media were determined by ELISA and normalized to cellular protein content. For the 48 h condition, conditioned medium collected after medium renewal at 24 h represents secretion during the 24–48 h interval. IL-6 and MCP-1 were also examined in HepG2 conditioned media but remained below the reliable quantification range. Data are presented as mean ± SEM. Statistical significance was evaluated against the corresponding BSA control. * *p* < 0.05, ** *p* < 0.01, *** *p* < 0.001; ns, not significant.

Inflammatory signaling was assessed via p-NF-κB p65 (Ser536), COX-2, TNF-α, and IL-1β protein levels at 24 h and 48 h (Figure 8). Different inflammatory markers showed distinct response patterns across FFA conditions, indicating that inflammatory outputs varied according to both marker type and fatty acid composition rather than converging into a single dominant inflammatory state.

NF-κB signaling (p-NF-κB/NF-κB ratio; Figure 8): At 24 h, O2P1 showed the highest phosphorylation ratio (7.63 ± 0.25; *p* < 0.001 vs. BSA), followed by OA (4.57 ± 0.59; *p* < 0.01). PA (3.12 ± 0.70), O1P1, O3P2, and O1P2 (1.32 ± 0.55) did not differ significantly from BSA, indicating that significantly elevated NF-κB phosphorylation at 24 h was restricted to OA and O2P1. At 48 h, none of the treatment groups differed significantly from BSA.

COX-2 protein abundance (Figure 8): O1P2 showed the highest COX-2 levels (2.17 ± 0.22), followed by PA (1.79 ± 0.12) and O1P1 (1.74 ± 0.08), whereas O2P1 (1.52 ± 0.11) exhibited a comparatively modest increase despite strong NF-κB activation. COX-2 responses did not parallel NF-κB phosphorylation and were prominent in several PA-containing conditions.

TNF-α protein abundance (Figure 8): TNF-α levels were similarly elevated in O2P1 (2.35 ± 0.02) and O3P2 (2.33 ± 0.05), followed by O1P2 (2.13 ± 0.11) and O1P1 (1.93 ± 0.21), while OA and PA showed lower levels (1.17 and 1.56, respectively). These findings indicate that TNF-α induction does not directly parallel NF-κB phosphorylation and instead reflects an additional inflammatory dimension shaped by OA:PA composition.

IL-1β protein abundance (Figure 8): At 24 h, IL-1β was significantly elevated in O1P2 (1.50 ± 0.11; *p* < 0.01), O3P2 (1.49 ± 0.07; *p* < 0.01), and O2P1 (1.43 ± 0.05; *p* < 0.05), whereas O1P1 (1.33 ± 0.07), OA (1.03 ± 0.07), and PA (1.26 ± 0.08) did not differ significantly from BSA. At 48 h, IL-1β did not differ significantly from BSA in any condition.

Collectively, these findings support the conclusion that inflammatory responses under FFA overload are multidimensional and differ according to both the selected marker and OA:PA ratio.

Secreted IL-8 was measured in HepG2 conditioned media by ELISA (Figure 8C). At 24 h, IL-8 concentrations were significantly higher than BSA (14.63 ± 0.06 pg/µg protein) in OA (19.16 ± 0.08; *p* < 0.001), PA (17.34 ± 0.33; *p* < 0.01), O1P1 (18.77 ± 0.39; *p* < 0.001), O2P1 (19.13 ± 0.54; *p* < 0.001), and O3P2 (20.15 ± 1.07; *p* < 0.001), whereas O1P2 (14.62 ± 0.67 pg/µg protein) did not differ significantly from BSA. At 48 h, PA (5.08 ± 0.25; *p* < 0.001), O1P1 (6.73 ± 0.07; *p* < 0.001), O2P1 (8.18 ± 0.25; *p* < 0.001), O3P2 (9.06 ± 0.43; *p* < 0.05), and O1P2 (7.78 ± 0.60; *p* < 0.01) fell significantly below BSA (10.55 ± 0.14 pg/µg protein), whereas OA (11.81 ± 0.37) did not differ significantly from BSA. Because the medium was renewed at 24 h, the 48 h conditioned medium represents secretion during the 24–48 h interval rather than cumulative 48 h secretion. IL-6 and MCP-1 were also measured in HepG2 conditioned media but remained below the reliable quantification range of the respective assays and are therefore not reported quantitatively.

### 3.8. Autophagy-Related Markers and ER Stress Responses Showed Limited Changes Under Acute FFA Exposure

**Figure 9:**
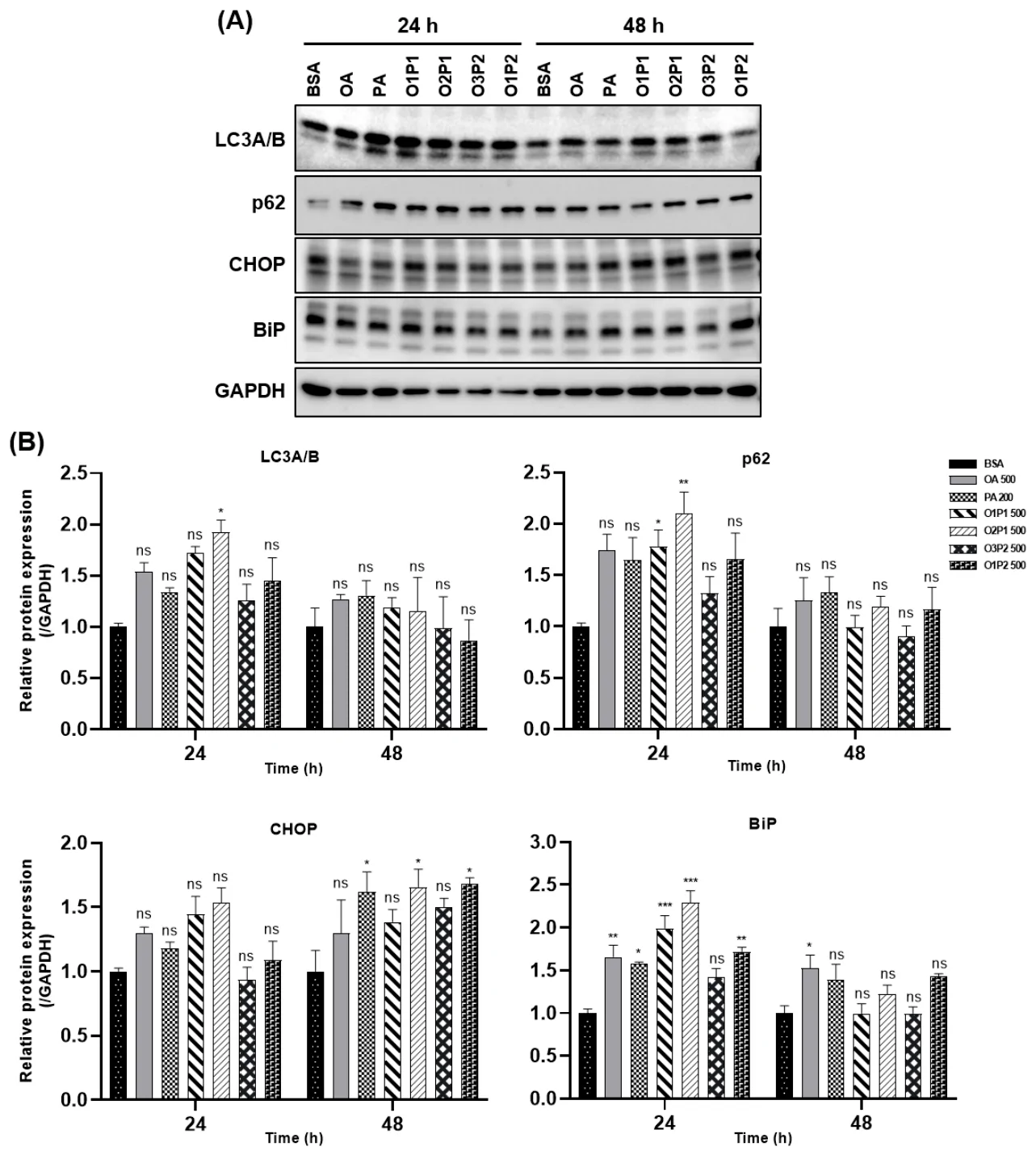
Effects of OA:PA composition on ER-stress- and autophagy-related proteins in HepG2 cells. (**A**) Representative Western blot images of LC3A/B, p62, CHOP, and BiP in HepG2 cells treated with OA (500 µM), PA (200 µM), or OA:PA mixtures (500 µM) for 24 h and 48 h. GAPDH was used as a loading control. (**B**) Quantification of relative protein expression. Data are presented as mean ± SEM and expressed relative to the corresponding BSA control. * *p* < 0.05, ** *p* < 0.01, *** *p* < 0.001; ns, not significant.

Autophagy-related and ER stress markers were assessed to determine their contribution to ratio-dependent phenotypic differences (Figure 9). At 24 h, LC3A/B protein levels were significantly elevated only in O2P1 (1.93 ± 0.12; *p* < 0.05); OA (1.54 ± 0.09), PA (1.34 ± 0.04), and O1P1 (1.72 ± 0.06) did not differ significantly from BSA (Con = 1.000). p62 levels were significantly increased in O1P1 (1.78 ± 0.16; *p* < 0.05) and O2P1 (2.10 ± 0.21; *p* < 0.01). Although several autophagy-related proteins showed statistically detectable alterations, the magnitude of change remained modest, and interpretation is limited in the absence of autophagic flux assessment using pharmacological tools such as bafilomycin A1. Therefore, these observations reflect alterations in marker protein abundance and should not be interpreted as definitive evidence of increased or impaired autophagic activity.

BiP levels were significantly elevated at 24 h in OA (1.66 ± 0.13), PA (1.58 ± 0.02), O1P1 (1.99 ± 0.15), and O2P1 (2.29 ± 0.14), indicating activation of ER stress-associated responses during early FFA exposure across multiple FFA conditions (Figure 9).

CHOP did not differ significantly from BSA at 24 h in any condition (Figure 9), including O2P1 (1.53 ± 0.12) and O1P1 (1.45 ± 0.14; both ns). At 48 h, CHOP was significantly elevated in PA (1.62 ± 0.15; *p* < 0.05), O2P1 (1.66 ± 0.14; *p* < 0.05), and O1P2 (1.68 ± 0.05; *p* < 0.05), whereas O3P2 (1.50 ± 0.07) and O1P1 (1.39 ± 0.10) did not reach significance, suggesting that CHOP-associated ER stress responses emerged later and were not uniform across conditions.

Taken together, these findings indicate that ER stress-related responses, reflected by BiP and CHOP expression, were detectable from 24 h and occurred across multiple FFA conditions rather than being restricted to PA-dominant exposure. Because autophagic flux was not directly assessed, the concurrent elevation of LC3A/B, p62, BiP, and CHOP should be interpreted as activation of stress-responsive pathways rather than confirmed autophagic dysfunction or fully resolved ER stress mechanisms.

### 3.9. Integrated Phenotypic Positioning Map: Each OA:PA Ratio Generates a Distinct Hepatocellular State

**Figure 10:**
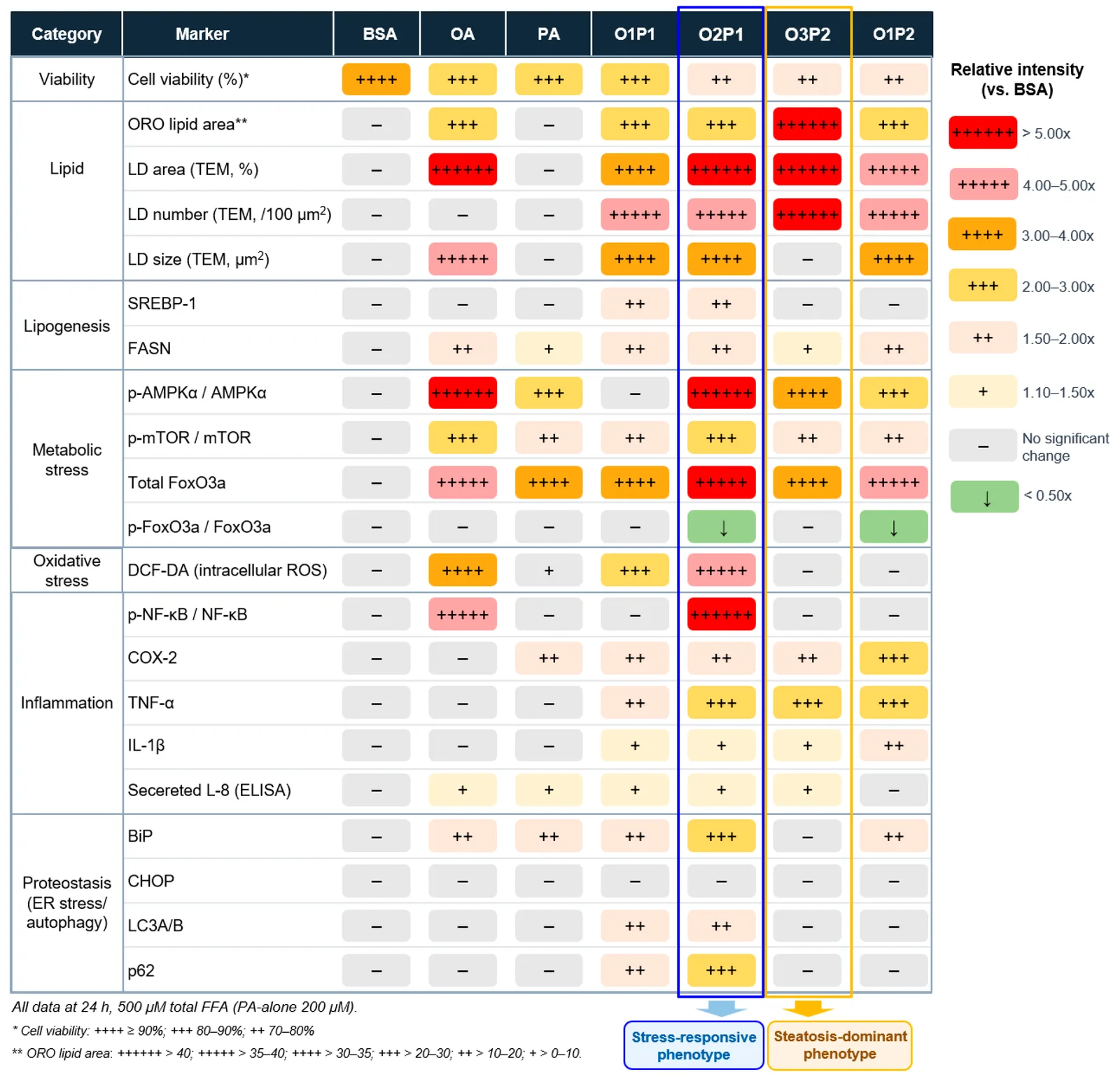
Integrated heatmap of OA:PA ratio responses relative to the BSA control at 500 μM total FFA, except for PA alone (200 μM). Only statistically significant changes versus BSA are color-coded; non-significant changes are shown in gray. Relative increases were categorized as +, 1.10–1.50-fold; ++, 1.50–2.00-fold; +++, 2.00–3.00-fold; ++++, 3.00–4.00-fold; +++++, 4.00–5.00-fold; and ++++++, >5.00-fold. Downward arrows indicate significant decreases. Cell viability and Oil Red O (ORO) lipid area were classified using the separate thresholds indicated below the heatmap. O2P1 and O3P2 are highlighted as representative stress-responsive and steatosis-dominant phenotypes, respectively. Colored category labels are used for visual grouping of the measured response domains; heatmap cell colors indicate the magnitude of statistically significant changes according to the scale shown in the figure.

The multi-endpoint dataset was integrated into a phenotypic positioning map (Figure 10) to summarize the distinct hepatocellular states generated under different OA:PA ratio conditions. Rather than forming a linear continuum from steatosis to lipotoxicity, different OA:PA ratios produced discrete phenotypic states characterized by distinct combinations of lipid storage, stress adaptation, and inflammatory responses.

(I) Steatosis phenotype (representative condition: O3P2): O3P2 was characterized by pronounced lipid accumulation with a high LD burden (ORO: 30.47 ± 3.16 at 500 µM; Figure 3 and Figure 4; LD number: 69.59 ± 10.05/100 µm^2^; mean LD size: 0.298 ± 0.036 µm^2^) and dispersed microvesicular LD distribution, accompanied by significant AMPK-associated signaling but no significant NF-κB activation relative to BSA. O3P2 may serve as a representative condition for studies focused on lipid storage and LD morphology.

(II) Pronounced stress-responsive phenotype with selected MASH-relevant features (representative condition: O2P1): This state was characterized by substantial lipid accumulation (ORO: 28.71 ± 1.67; LD area: 14.66 ± 1.44%) together with elevated intracellular DCF-DA fluorescence (4.23-fold vs. BSA; *p* < 0.001; Figure 5), enhanced AMPK activation (p-AMPKα/AMPKα: 6.97 ± 0.13; Figure 7), elevated inflammatory signaling (p-NF-κB/NF-κB: 7.63 ± 0.25; Figure 8), and measurable IL-8 secretion in conditioned medium (Figure 8C), while maintaining experimental viability. These features represent a pronounced stress-responsive phenotype rather than a direct model of MASH. O2P1 may serve as a representative condition for investigating selected MASH-relevant stress-responsive signaling under acute FFA exposure.

(III) Lipotoxic phenotype (representative condition: PA): This state was characterized by greater cytotoxicity accompanied by relatively limited neutral lipid storage. O1P2, a PA-enriched condition that maintained viability and ORO accumulation comparable to O3P2, additionally showed elevated COX-2 abundance (2.17 ± 0.22; Figure 8), increased TNF-α and IL-1β protein expression, and CHOP induction at 24 h and/or 48 h (Figure 9), and may serve as a more narrowly defined representative condition for PA-enriched inflammatory/ER-stress-associated studies. These findings suggest that PA-enriched conditions favor stress-associated and inflammatory responses over efficient intracellular lipid sequestration and may serve as representative conditions for lipotoxicity- and ER stress-associated studies.

### 3.10. Selected OA:PA-Associated Responses Were Evaluated in SNU-449 Cells

To evaluate whether OA:PA ratio-dependent phenotypic differences extend beyond HepG2 cells, selected responses were examined in SNU-449 cells, an independent hepatocellular carcinoma cell line, under matched OA (500 µM), PA (200 µM), O2P1 (500 µM total FFA), and O3P2 (500 µM total FFA) conditions at 24 h (Supplementary Figures S2–S4). Cell viability was significantly reduced relative to BSA in OA (84.7%; *p* < 0.001), PA (88.4%; *p* < 0.001), and O2P1 (96.7%; *p* < 0.01), whereas O3P2 (99.3%) did not differ significantly from BSA (Supplementary Figure S2A). ORO staining showed significantly increased lipid accumulation in OA (128.7-fold vs. BSA; *p* < 0.001), O2P1 (50.8-fold; *p* < 0.001), and O3P2 (40.9-fold; *p* < 0.001), whereas PA (2.9-fold) did not reach significance (Supplementary Figure S2B). In contrast to the HepG2 pattern, DCF-DA fluorescence was significantly elevated in PA (2.09-fold; *p* < 0.05), O2P1 (2.14-fold; *p* < 0.01), and O3P2 (2.03-fold; *p* < 0.05), while OA (0.70-fold) did not differ significantly from BSA (Supplementary Figure S3A). Secreted IL-8 was significantly elevated in all FFA-treated conditions relative to BSA (1.06 ng/µg protein), with OA (1.42; *p* < 0.001), O2P1 (1.36; *p* < 0.001), O3P2 (1.22; *p* < 0.05), and PA (1.21 ng/µg protein; *p* < 0.05) (Supplementary Figure S3B); unlike in HepG2 conditioned media, MCP-1 and IL-6 were also quantifiable in SNU-449 conditioned media, with MCP-1 significantly elevated in PA and O2P1 and IL-6 significantly elevated in PA, O2P1, and O3P2 (Supplementary Figure S3C,D). At the molecular level, SREBP-1 and total AMPKα were significantly reduced in OA relative to BSA (*p* < 0.05 and *p* < 0.01, respectively), while PA, O2P1, and O3P2 did not differ significantly from BSA for either marker; p-AMPKα and the p-AMPKα/AMPKα ratio were significantly elevated in OA (*p* < 0.001) and O2P1 (*p* < 0.05), with PA and O3P2 showing no significant change (Supplementary Figure S4). BAX, BCL-2, and the BAX/BCL-2 ratio did not differ significantly across conditions. Although BAX abundance changed in several HepG2 conditions, neither cell line showed a significant shift in the BAX/BCL-2 ratio. Together, these findings indicate that composition-dependent responses were also observed in SNU-449 cells, but with a distinct response hierarchy from HepG2, particularly for oxidative stress signaling, underscoring cell-line-dependent variation in the magnitude and pattern of these responses.

## 4. Discussion

The present study demonstrates that different OA:PA ratios generate discrete hepatocellular states rather than representing a simple linear transition from steatosis to lipotoxicity. Four empirical observations collectively define this phenotypic landscape: (I) OA predominantly promotes neutral lipid storage; (II) PA preferentially induces lipotoxic stress with comparatively limited lipid accumulation; (III) O3P2 maximizes steatosis and increases LD number with a relatively smaller mean LD size than OA-rich conditions; and (IV) O2P1 co-features steatosis together with metabolic and inflammatory stress responses, reproducing selected molecular characteristics associated with MASH-related cellular stress.

An important motivation for this study was the considerable heterogeneity in OA:PA compositions used across FFA-based hepatocyte models and the limited experimental basis for treating these ratios as interchangeable [4,10,11]. Our findings demonstrate that OA:PA ratio should be considered an independent experimental variable. Even under a standardized total FFA load, modest changes in OA:PA composition produced distinct phenotypic states: O2P1 preferentially exhibited pronounced oxidative and AMPK/NF-κB-associated stress-responsive signaling, whereas O3P2 favored lipid storage with a high LD burden and comparatively less pronounced stress signaling. Thus, the choice of OA:PA ratio can qualitatively influence the biological phenotype observed and, consequently, the interpretation of an FFA-based hepatocyte model.

To address the limitation of using a single hepatoma cell line, selected OA:PA-associated responses were additionally evaluated in SNU-449 cells, an independent hepatocellular carcinoma cell line with distinct metabolic characteristics (Section 3.10; Supplementary Figures S2–S4). SNU-449 cells exhibited OA:PA ratio-dependent changes in lipid accumulation, oxidative responses, and cytokine secretion; however, the relative response hierarchy differed from that observed in HepG2 cells, most notably for DCF-DA-based oxidative stress signaling. These findings highlight cell-line-dependent metabolic and inflammatory responses and support the concept that OA:PA ratio effects are modulated by the metabolic context of the host cell line. Full translational validation in primary human hepatocytes remains an important future direction.

Previous in vitro hepatic steatosis studies have generally relied on single FFA species or selected OA:PA mixtures without systematic comparison of composition-dependent effects [4,10,11]. Eynaudi et al. [13] characterized OA/PA effects on LD–mitochondria interactions but employed only a single OA + PA mixture, while Gomez-Lechon et al. [11] included TEM assessment without quantitative evaluation of LD morphology or integrated molecular pathway profiling. The present study extends these approaches through systematic comparison across four OA:PA ratios with concurrent TEM-based LD morphometrics (Figure 3 and Figure 4) and multi-pathway molecular profiling (Figure 5, Figure 6, Figure 7, Figure 8 and Figure 9), and integrated phenotypic positioning (Figure 10).

The contrasting effects of OA and PA reflect their fundamentally different intracellular handling. OA is efficiently esterified into triglycerides and sequestered within LDs, thereby reducing exposure of excess FFAs to stress-responsive pathways [5,6]. Consistent with this, OA-rich conditions generally showed greater ORO signals and larger electron-lucent LDs (Figure 3 and Figure 4). In contrast, PA was associated with lower lipid accumulation despite greater cytotoxicity (Figure 2 and Figure 3), consistent with both recent and foundational observations linking saturated FFA exposure to lipotoxicity, ER stress, and inflammatory responses [7,8,21,22].

Interestingly, TEM analysis suggested that OA:PA composition influenced LD morphology in addition to total lipid accumulation. OA-rich conditions were associated with relatively larger LD structures, whereas O3P2 was distinguished by a high LD number and a relatively smaller mean LD size than OA-rich conditions (Figure 4). These observations suggest that OA:PA composition may influence intracellular lipid organization independently of total lipid accumulation. Previous studies have reported that monounsaturated fatty acids preferentially support neutral lipid storage and LD expansion, whereas saturated fatty acids may alter intracellular lipid partitioning and favor accumulation of smaller LD structures [5,23,24]. However, because LD fusion dynamics and intracellular lipid remodeling were not directly evaluated in the present study, these findings should be interpreted as morphological observations rather than direct mechanistic evidence.

Importantly, the molecular behavior of O2P1 relative to O3P2 cannot be explained simply by absolute PA abundance. Although O3P2 contained a higher absolute PA concentration than O2P1 at equivalent total FFA exposure, O2P1 exhibited substantially greater AMPK phosphorylation and NF-κB activation (Figure 7 and Figure 8). Conditions containing equal or greater PA proportions, including O1P1, O1P2, and PA alone, did not exhibit comparable stress-responsive signaling.

These findings indicate that distinct OA:PA compositions were associated with divergent hepatocellular phenotypes rather than a single graded response (Figure 7, Figure 8 and Figure 10). Relative to BSA, OA alone was associated with substantial lipid storage together with metabolic and inflammatory stress-responsive signaling. Among the mixed conditions, O2P1 combined comparable lipid accumulation with the strongest AMPK- and NF-κB-associated signaling observed in this study, whereas O3P2 combined comparable or greater lipid accumulation with only modest AMPK-associated signaling and no significant NF-κB activation relative to BSA. This divergence indicates that OA:PA composition, rather than PA content alone, was associated with the resulting hepatocellular phenotype.

The divergent LD morphologies observed across conditions provide an additional structural dimension to this interpretation. Whereas OA-rich conditions were associated with relatively enlarged LD structures, O3P2 exhibited increased LD number together with a relatively smaller mean LD size than OA-rich conditions despite extensive lipid accumulation (Figure 4), supporting the concept that lipid storage and stress responsiveness may diverge across OA:PA compositions.

**Figure 11:**
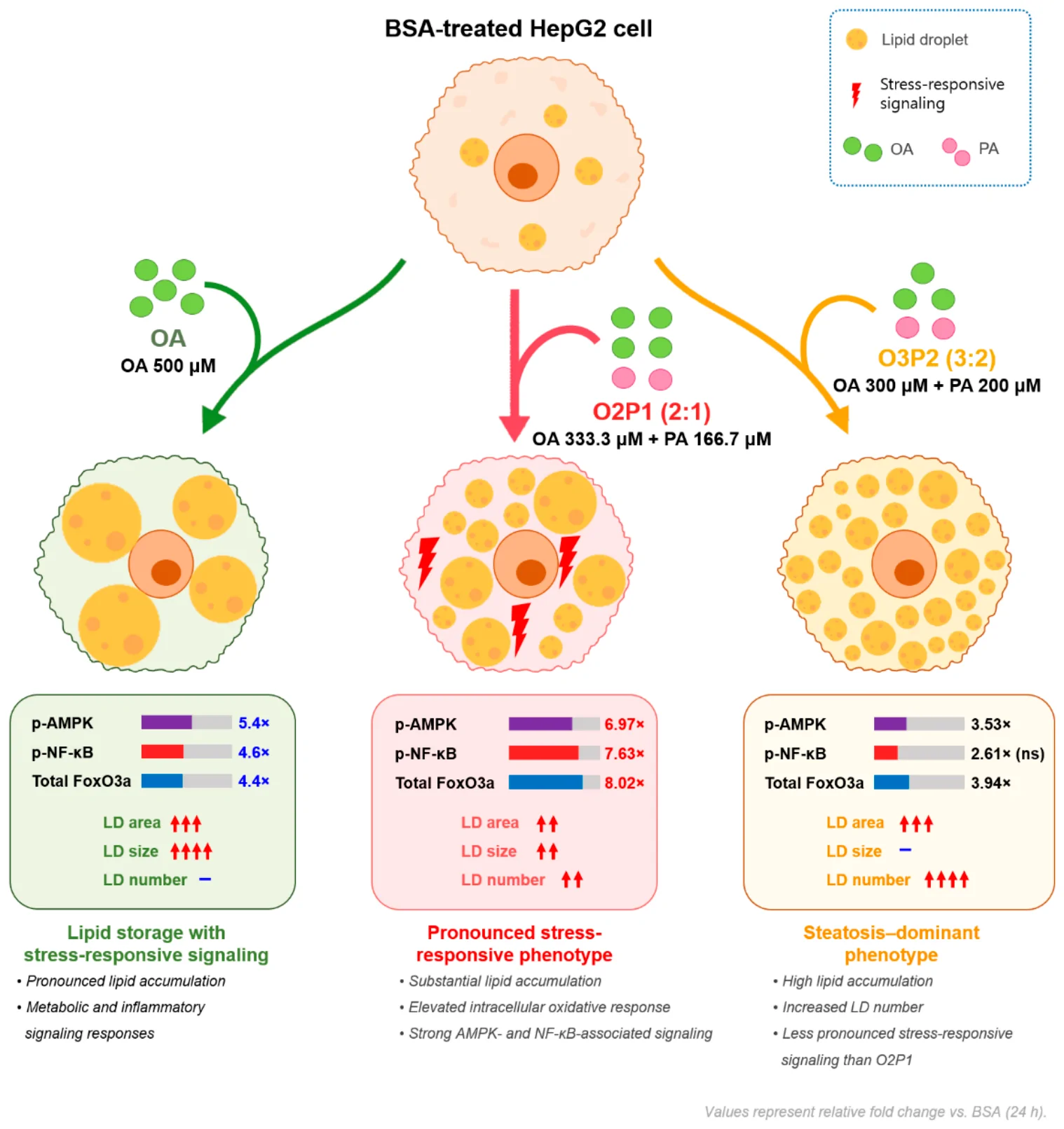
Conceptual model of OA:PA composition-dependent hepatocellular phenotype divergence in HepG2 cells. Relative to BSA-treated HepG2 controls, 24 h exposure to OA alone (500 μM), O2P1 (OA:PA = 2:1; OA 333.3 μM + PA 166.7 μM), or O3P2 (OA:PA = 3:2; OA 300 μM + PA 200 μM) produced distinct hepatocellular phenotypes. OA treatment was characterized by pronounced lipid storage accompanied by metabolic and inflammatory stress-responsive signaling. O2P1 exhibited substantial lipid accumulation together with an elevated intracellular oxidative response and strong AMPK- and NF-κB-associated signaling, representing a pronounced stress-responsive phenotype. In contrast, O3P2 exhibited extensive lipid accumulation with a marked increase in lipid-droplet number but comparatively less pronounced stress-responsive signaling, representing a steatosis-dominant phenotype. Values shown for p-AMPK, p-NF-κB, and total FoxO3a represent fold changes at 24 h relative to the BSA control; phosphorylated-protein values correspond to phospho/total protein ratios. LD area, LD size, and LD number schematically summarize TEM-derived quantitative changes relative to BSA; upward arrows indicate increases, with a greater number of arrows representing a larger relative increase, whereas “–” indicates no significant change. This schematic represents an integrated conceptual interpretation of the experimental findings and does not imply direct mechanistic causality or synergistic interaction between OA and PA.

To facilitate interpretation of the integrated phenotypic responses observed across OA:PA conditions, Figure 11 presents a conceptual model summarizing three representative hepatocellular states arising from a common BSA-treated baseline. OA alone, O2P1, and O3P2 generated distinct combinations of lipid storage and stress-responsive signaling, illustrating that OA:PA composition was associated with divergent rather than sequential hepatocellular phenotypes. Importantly, Figure 11 is intended as a conceptual synthesis of the experimental observations and should not be interpreted as evidence of direct mechanistic causality.

The behavior of O2P1 merits particular discussion. O2P1 produced substantial lipid accumulation while simultaneously exhibiting enhanced AMPK phosphorylation and inflammatory signaling (Figure 3, Figure 7 and Figure 8). These findings suggest that O2P1 may represent a pronounced stress-responsive lipid overload state rather than a purely steatotic condition (Figure 7, Figure 8 and Figure 10). Importantly, this interpretation does not imply that O2P1 fully recapitulates MASH pathology, but rather that it reproduces selected molecular features associated with MASH-relevant cellular stress.

Another notable observation was the behavior of FoxO3a (Figure 7). Total FoxO3a abundance increased significantly across all FFA conditions at 24 h and reached the highest level in O2P1, whereas phosphorylation did not increase proportionally, resulting in a p-FoxO3a/FoxO3a ratio that was significantly reduced in OA, O1P1, O2P1, and O3P2 and did not differ significantly from BSA in PA and O1P2. At 48 h, this pattern was no longer uniform: the p-FoxO3a/FoxO3a ratio remained significantly elevated relative to BSA only in O1P1 and O1P2, while other conditions did not differ significantly from BSA. These findings suggest that FoxO3a regulation under acute FFA overload involves changes in both total protein abundance and phosphorylation state. However, because nuclear localization and downstream transcriptional targets were not evaluated, the functional significance of these changes remains speculative.

An additional conceptual contribution of this study is the demonstration that inflammatory signaling under FFA overload is multidimensional rather than uniformly regulated. NF-κB phosphorylation peaked in O2P1, whereas COX-2 and IL-1β exhibited distinct response patterns across conditions (Figure 8). These marker-specific differences indicate that inflammatory responses cannot be reduced to a single dominant condition and emphasize that model selection should be guided by the biological process of interest (Figure 10).

Among these markers, IL-8 (CXCL8) was of particular interest as a secreted inflammatory mediator because NF-κB activation has been shown to promote IL-8 secretion from FFA-stressed human hepatocytes, including HepG2 cells [18]. Functionally, IL-8 is a potent neutrophil chemoattractant; thus, its quantification in conditioned medium provides an extracellular chemokine readout that complements the intracellular inflammatory markers (NF-κB, COX-2, TNF-α, and IL-1β). In the present study, increased IL-8 secretion at 24 h in several FFA-treated conditions, including O2P1, indicates an altered hepatocyte-derived secretory inflammatory response. However, neutrophil chemotaxis or other immune-cell functional responses were not directly assessed; therefore, the IL-8 data should be interpreted as evidence of altered chemokine secretion rather than direct demonstration of immune-cell recruitment or activation.

The temporal dissociation observed between molecular signaling and structural lipid accumulation provides another important insight. Whereas signaling markers including AMPK and NF-κB showed their strongest responses at 24 h (Figure 7 and Figure 8), TEM-derived LD area and/or number remained elevated or increased at 48 h, while several signaling markers no longer differed significantly from BSA and ORO responses showed a different temporal pattern (Figure 3 and Figure 4). This temporal separation suggests that early molecular stress signaling and later structural lipid storage may represent partially independent phases of adaptation to acute FFA overload.

Several limitations should be acknowledged. First, HepG2 cells are derived from hepatoblastoma and exhibit lower xenobiotic-metabolizing capacity, including reduced cytochrome P450 activity, compared with primary human hepatocytes [4,25]. Secondary evaluation in SNU-449 cells (Section 3.10; Supplementary Figures S2–S4) demonstrated composition-dependent responses, although the magnitude and relative hierarchy differed from those observed in HepG2 cells, underscoring the need for validation in primary human hepatocytes. Second, autophagic flux was not directly evaluated using bafilomycin A1; changes in LC3A/B and p62 should not be interpreted as definitive evidence of altered autophagic activity [26]. Third, mitochondrial function, including membrane potential and respiratory activity, was not directly assessed; TEM-based observations should be interpreted as ultrastructural alterations rather than functional evidence of mitochondrial dysfunction. Fourth, BAX increased in several conditions, whereas BCL-2 and the BAX/BCL-2 ratio were not significantly altered; therefore, the data did not support a robust apoptotic shift under the tested conditions (Supplementary Figure S1). Fifth, while IL-8 secretion was quantifiable in HepG2 conditioned medium, IL-6 and MCP-1 remained below reliable quantification limits, and functional validation using macrophage or hepatic stellate cell co-culture systems was not performed; this represents an important future direction. Sixth, the study focused on acute exposure periods (24–48 h) and did not evaluate fibrosis-associated responses, insulin resistance, or chronic steatosis [27].

Rather than proposing a single optimal OA:PA ratio, the present study demonstrates that selection of in vitro lipid overload conditions should be guided by the intended phenotypic dimension under investigation (Figure 10). Collectively, these findings support a phenotype-oriented framework for selecting OA:PA conditions in hepatocyte-based in vitro modeling and may facilitate more consistent design and interpretation of mechanistic and pharmacological studies. These findings support phenotype-oriented ratio selection rather than the routine adoption of a single OA:PA formulation based primarily on historical convention.

## 5. Conclusions

Different OA:PA ratios generated discrete hepatocellular states rather than representing a linear steatosis-to-lipotoxicity continuum. O3P2 produced a steatosis-dominant phenotype characterized by maximal lipid accumulation, increased LD number, and less pronounced oxidative or metabolic stress signaling. O2P1 generated a pronounced stress-responsive phenotype with selected MASH-relevant molecular features, co-featuring substantial lipid accumulation, elevated intracellular ROS, enhanced AMPK and NF-κB signaling, and measurable IL-8 secretion. Selected OA:PA-associated responses were additionally evaluated in SNU-449 cells, revealing OA:PA ratio-dependent but cell-line-specific responses. These findings do not propose O2P1 as a comprehensive MASH model, but rather support a phenotype-oriented framework for selecting OA:PA conditions according to the specific biological process under investigation. This approach may facilitate more rational experimental design and interpretation in hepatocyte-based in vitro MASLD/MASH research.

## Figures and Tables

**Table 1:** Composition of representative FFA treatment conditions used for downstream phenotypic and molecular analyses.

Group	OA:PA Ratio	OA (μM)	PA (μM)	Total FFA (μM)	OA-BSA (μL)	PA-BSA (μL)	Vehicle-BSA (μL)	Medium (μL)	Final Vol. (μL)	Final BSA (mg/mL)
BSA	—	0	0	0	0	0	20	80	100	19
OA	100% OA	500	0	500	10	0	10	80	100	19
PA	100% PA	0	200	200	0	4	16	80	100	19
O1P1	1:1	250	250	500	5	5	10	80	100	19
O2P1	2:1	333.30	166.70	500	6.67	3.33	10	80	100	19
O3P2	3:2	300	200	500	6	4	10	80	100	19
O1P2	1:2	166.70	333.30	500	3.33	6.67	10	80	100	19

OA–BSA, PA–BSA, and FFA-free vehicle-BSA solutions contained the same BSA concentration [9.5% (*w*/*v*)]. For all treatment groups, the combined volume of OA–BSA, PA–BSA, and vehicle-BSA solution was fixed at 20 μL and culture medium at 80 μL per 100 μL final treatment volume. Thus, the final BSA concentration was maintained at 19 mg/mL [1.9% (*w*/*v*)] in all treatment and control conditions. Volumes shown are based on a 100 μL final treatment volume and were scaled proportionally for other culture formats.

## Data Availability

The original contributions presented in this study are included in the article. Further inquiries can be directed to the corresponding authors.

## Supplementary Materials

The following supporting information can be downloaded at https://www.mdpi.com/article/10.3390/cells15191774/s1, Figure S1: Effects of OA:PA composition on apoptosis-related protein expression in HepG2 cells; Figure S2: Selected viability and lipid-accumulation responses to OA:PA treatment in SNU-449 cells; Figure S3: Oxidative and cytokine responses to OA:PA treatment in SNU-449 cells; Figure S4: Selected lipogenic and AMPK-associated signaling responses to OA:PA treatment in SNU-449 cells.

## Abbreviations

The following abbreviations are used in this manuscript:

AMPK	AMP-activated protein kinase
ANOVA	analysis of variance
BAX	BCL-2-associated X protein
BCA	bicinchoninic acid
BCL-2	B-cell lymphoma 2
BiP	binding immunoglobulin protein
BSA	bovine serum albumin
CHOP	C/EBP homologous protein
COX-2	cyclooxygenase-2
CYP	cytochrome P450
DCF-DA	2′,7′-dichlorofluorescin diacetate
DMEM	Dulbecco’s modified Eagle’s medium
DMSO	dimethyl sulfoxide
ELISA	enzyme-linked immunosorbent assay
ER	endoplasmic reticulum
FASN	fatty acid synthase
FBS	fetal bovine serum
FFA	free fatty acid
FoxO3a	forkhead box O3a
GAPDH	glyceraldehyde-3-phosphate dehydrogenase
HBSS	Hank’s balanced salt solution
HRP	horseradish peroxidase
IL-1β	interleukin-1 beta
IL-6	interleukin-6
IL-8	interleukin-8
LC3A/B	microtubule-associated protein 1A/1B-light chain 3A/B
LD	lipid droplet
MASH	metabolic dysfunction-associated steatohepatitis
MASLD	metabolic dysfunction-associated steatotic liver disease
MCP-1	monocyte chemoattractant protein-1
mTOR	mechanistic target of rapamycin
NF-κB	nuclear factor kappa-light-chain-enhancer of activated B cells
OA	oleic acid
ORO	Oil Red O
p62/SQSTM1	sequestosome 1
PA	palmitic acid
PBS	phosphate-buffered saline
PVDF	polyvinylidene difluoride
RIPA	radioimmunoprecipitation assay
ROS	reactive oxygen species
SDS-PAGE	sodium dodecyl sulfate-polyacrylamide gel electrophoresis
SEM	standard error of the mean
SREBP-1	sterol regulatory element-binding protein 1
TBST	Tris-buffered saline with Tween-20
TEM	transmission electron microscopy
TNF-α	tumor necrosis factor-alpha
WST	water-soluble tetrazolium

## References

[1] Rinella M.E., Lazarus J.V., Ratziu V., Francque S.M., Sanyal A.J., Kanwal F., Romero D., Abdelmalek M.F., Anstee Q.M., Arab J.P. (2023). A multisociety Delphi consensus statement on new fatty liver disease nomenclature. J. Hepatol..

[2] Targher G., Valenti L., Byrne C.D. (2025). Metabolic Dysfunction–Associated Steatotic Liver Disease. N. Engl. J. Med..

[3] Friedman S.L., Neuschwander-Tetri B.A., Rinella M., Sanyal A.J. (2018). Mechanisms of NAFLD development and therapeutic strategies. Nat. Med..

[4] Kotlyarova A., Iskrina A., Kotlyarov S. (2026). The HepG2 Cell Line as a Model for Studying Metabolic Dysfunction-Associated Steatotic Liver Disease. Int. J. Mol. Sci..

[5] Listenberger L.L., Han X., Lewis S.E., Cases S., Farese R.V., Ory D.S., Schaffer J.E. (2003). Triglyceride accumulation protects against fatty acid-induced lipotoxicity. Proc. Natl. Acad. Sci. USA.

[6] Puri P., Baillie R.A., Wiest M.M., Mirshahi F., Choudhury J., Cheung O., Sargeant C., Contos M.J., Sanyal A.J. (2007). A lipidomic analysis of nonalcoholic fatty liver disease. Hepatology.

[7] Iturbe-Rey S., Maccali C., Arrese M., Aspichueta P., Oliveira C.P., Castro R.E., Lapitz A., Izquierdo-Sanchez L., Bujanda L., Perugorria M.J. (2025). Lipotoxicity-driven metabolic dysfunction-associated steatotic liver disease (MASLD). Atherosclerosis.

[8] Moliterni C., Vari F., Schifano E., Tacconi S., Stanca E., Friuli M., Longo S., Conte M., Salvioli S., Gnocchi D. (2024). Lipotoxicity of palmitic acid is associated with DGAT1 downregulation and abolished by PPARα activation in liver cells. J. Lipid Res..

[9] Karmakar E., Das N., Mukherjee B., Das P., Mukhopadhyay S., Roy S.S. (2023). Lipid-induced alteration in retinoic acid signaling leads to mitochondrial dysfunction in HepG2 and Huh7 cells. Biochem. Cell Biol..

[10] Ricchi M., Odoardi M.R., Carulli L., Anzivino C., Ballestri S., Lonardo A., Fantoni L.I., Loria P., Carulli N. (2009). Differential effect of oleic and palmitic acid on lipid accumulation and apoptosis in cultured hepatocytes. J. Gastroenterol. Hepatol..

[11] Gomez-Lechon M.J., Donato M.T., Martínez-Romero A., Jiménez N., Castell J.V., O’Connor J.E. (2007). A human hepatocellular in vitro model to investigate steatosis. Chem. Biol. Interact..

[12] Hetherington A.M., Sawyez C.G., Zilberman E., Stoianov A.M., Robson D.L., Borradaile N.M. (2016). Differential lipotoxic effects of palmitate and oleate in activated human hepatic stellate cells and epithelial hepatoma cells. Cell. Physiol. Biochem..

[13] Eynaudi A., Díaz-Castro F., Bórquez J.C., Bravo-Sagua R., Parra V., Lavandero S. (2021). Differential effects of oleic and palmitic acids on lipid droplet-mitochondria interaction in the hepatic cell line HepG2. Front. Nutr..

[14] Horton J.D., Goldstein J.L., Brown M.S. (2002). SREBPs: Activators of the complete program of cholesterol and fatty acid synthesis in the liver. J. Clin. Investig..

[15] Xie C., Liu K., Xie Y., Liu S., Ji B. (2024). Metabolism-related signalling pathways involved in the pathogenesis and development of metabolic dysfunction-associated steatotic liver disease. Clin. Res. Hepatol. Gastroenterol..

[16] Tikhanovich I., Cox J., Weinman S.A. (2013). Forkhead box class O transcription factors in liver function and disease. J. Gastroenterol. Hepatol..

[17] Elsharkawy A.M., Mann D.A. (2007). Nuclear factor-kappaB and the hepatic inflammation-fibrosis-cancer axis. Hepatology.

[18] Willy J.A., Young S.K., Stevens J.L., Masuoka H.C., Wek R.C. (2015). CHOP links endoplasmic reticulum stress to NF-κB activation in the pathogenesis of nonalcoholic steatohepatitis. Mol. Biol. Cell.

[19] Singh R., Kaushik S., Wang Y., Xiang Y., Novak I., Komatsu M., Tanaka K., Cuervo A.M., Czaja M.J. (2009). Autophagy regulates lipid metabolism. Nature.

[20] Venkatesan N., Doskey L.C., Malhi H. (2023). The Role of Endoplasmic Reticulum in Lipotoxicity during Metabolic Dysfunction–Associated Steatotic Liver Disease (MASLD) Pathogenesis. Am. J. Pathol..

[21] Holland W.L., Brozinick J.T., Wang L.P., Hawkins E.D., Sargent K.M., Liu Y., Narra K., Hoehn K.L., Knotts T.A., Siesky A. (2007). Inhibition of ceramide synthesis ameliorates glucocorticoid-, saturated-fat-, and obesity-induced insulin resistance. Cell Metab..

[22] Cazanave S.C., Wang X., Zhou H., Rahmani M., Grant S., Durrant D.E., Klaassen C.D., Yamamoto M., Sanyal A.J. (2014). Degradation of Keap1 activates BH3-only proteins Bim and PUMA during hepatocyte lipoapoptosis. Cell Death Differ..

[23] Krahmer N., Guo Y., Wilfling F., Hilger M., Lingrell S., Heger K., Newman H.W., Schmidt-Supprian M., Vance D.E., Mann M. (2011). Phosphatidylcholine synthesis for lipid droplet expansion is mediated by localized activation of CTP:phosphocholine cytidylyltransferase. Cell Metab..

[24] Krahmer N., Walther T.C., Farese R.V. (2025). The pathogenesis of hepatic steatosis in MASLD: A lipid droplet perspective. J. Clin. Investig..

[25] Westerink W.M.A., Schoonen W.G.E.J. (2007). Cytochrome P450 enzyme levels in HepG2 cells and cryopreserved primary human hepatocytes and their induction in HepG2 cells. Toxicol. In Vitro.

[26] Tanaka S., Hikita H., Tatsumi T., Sakamori R., Nozaki Y., Sakane S., Shiode Y., Nakabori T., Saito Y., Hiramatsu N. (2016). Rubicon inhibits autophagy and accelerates hepatocyte apoptosis and lipid accumulation in nonalcoholic fatty liver disease in mice. Hepatology.

[27] Ma Q., Liu K., Chang C., Wang L., Shen Z., Li J., Adu M., Lin Q., Huang H., Wu X. (2025). Metabolic dysfunction-associated steatotic liver disease: Pathogenesis, model and treatment (Review). Int. J. Mol. Med..

