# Supplementary material for: Distinct OA:PA Ratios Generate Discrete Hepatocellular Phenotypes in HepG2 Cells
Source: Cells. 2026 Sep 29;15(19):1774. doi: 10.3390/cells15191774 (PMC13664436; doi:10.3390/cells15191774)
Supplement: Supplementary file 1 [file cells-15-01774-s001.zip › cells-4424392-supplementary.pdf]

Supplementary Materials

Distinct OA:PA Ratios Generate Discrete Hepatocellular Phenotypes in HepG2 Cells

Hang Yeon Jeong, Ye Sol Han, Jiyeong Park, Han Byul Noh, Eunyoung Moon, Yang Hoon Huh

Figure S1

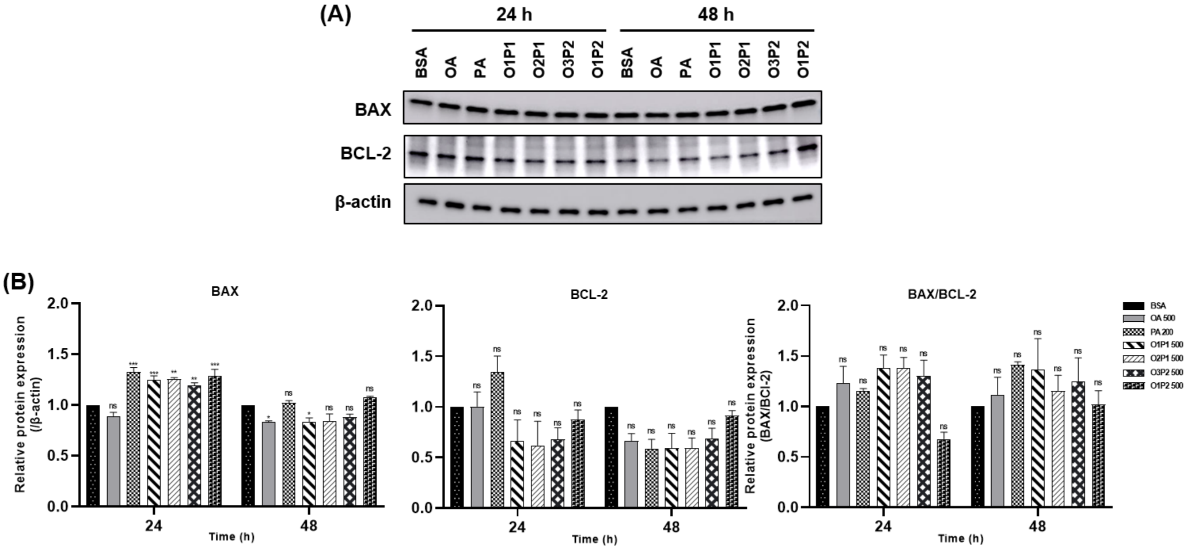

Figure S1. Effects of OA:PA composition on apoptosis-related protein expression in HepG2 cells. (A) Representative Western blot images of BAX and BCL-2 in HepG2 cells treated with BSA vehicle, OA (500 μM), PA (200 μM), or OA:PA mixtures (500 μM total FFA) for 24 or 48 h. β-Actin was used as a loading control. (B) Densitometric quantification of BAX and BCL-2 normalized to β-actin and the corresponding BAX/BCL-2 ratio. Data are presented as mean ± SEM (n = 3 independent biological replicates) and expressed relative to the corresponding BSA control. *$p < 0.05$, **$p < 0.01$, ***$p < 0.001$ vs. BSA; ns, not significant.

Figure S2

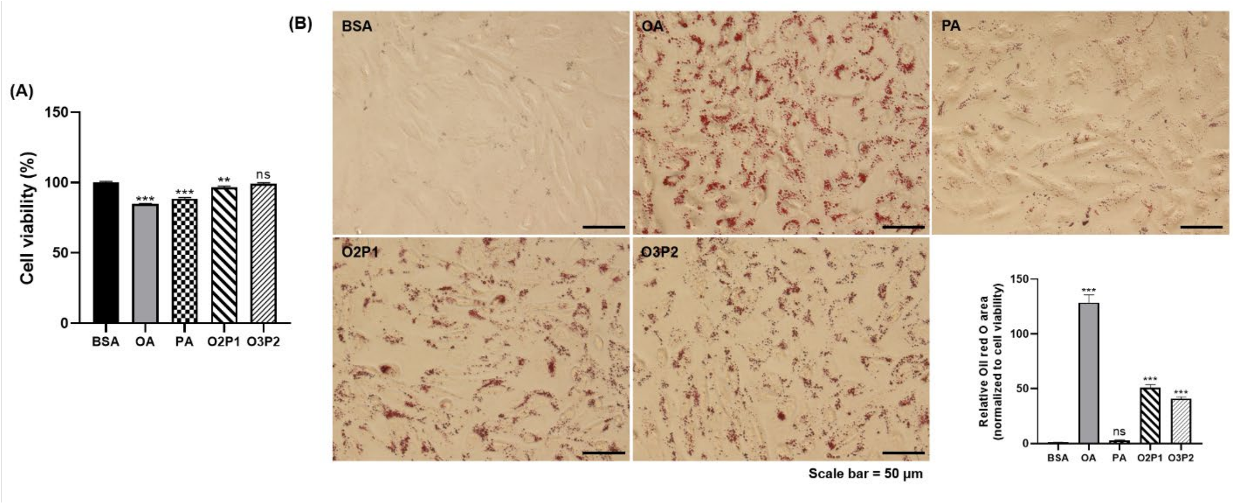

Figure S2. Selected viability and lipid-accumulation responses to OA:PA treatment in SNU-449 cells. SNU-449 cells were treated for 24 h with BSA vehicle, OA (500 μM), PA (200 μM), O2P1, or O3P2 (500 μM total FFA). (A) Cell viability was assessed using the WST assay. (B) Representative Oil Red O (ORO)-stained images and quantification of ORO-positive lipid accumulation normalized to cell viability. Data are presented as mean ± SEM. $^{**}p < 0.01$, $^{***}p < 0.001$ vs. BSA; ns, not significant. Scale bar = 50 μm.

Figure S3

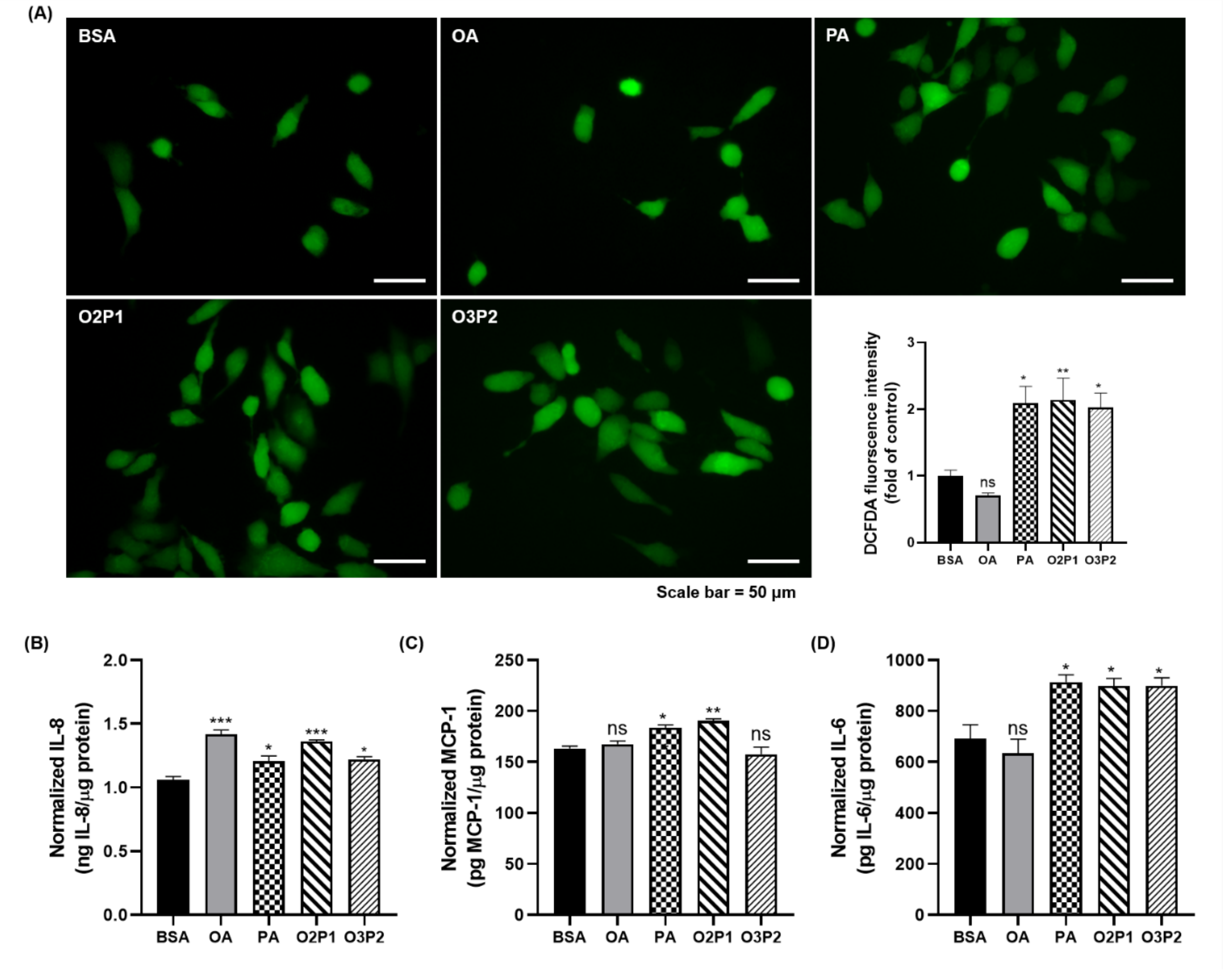

Figure S3. Oxidative and cytokine responses to OA:PA treatment in SNU-449 cells. SNU-449 cells were treated for 24 h with BSA vehicle, OA (500 µM), PA (200 µM), O2P1, or O3P2 (500 µM total FFA). (A) Representative DCF-DA fluorescence images and quantification of intracellular DCF-associated fluorescence expressed relative to the BSA control. Conditioned media were analyzed by ELISA for (B) IL-8, (C) MCP-1, and (D) IL-6, and cytokine concentrations were normalized to total cellular protein content. Data are presented as mean ± SEM. *$p < 0.05$, **$p < 0.01$, ***$p < 0.001$ vs. BSA; ns, not significant. Scale bar = 50 µm.

Figure S4

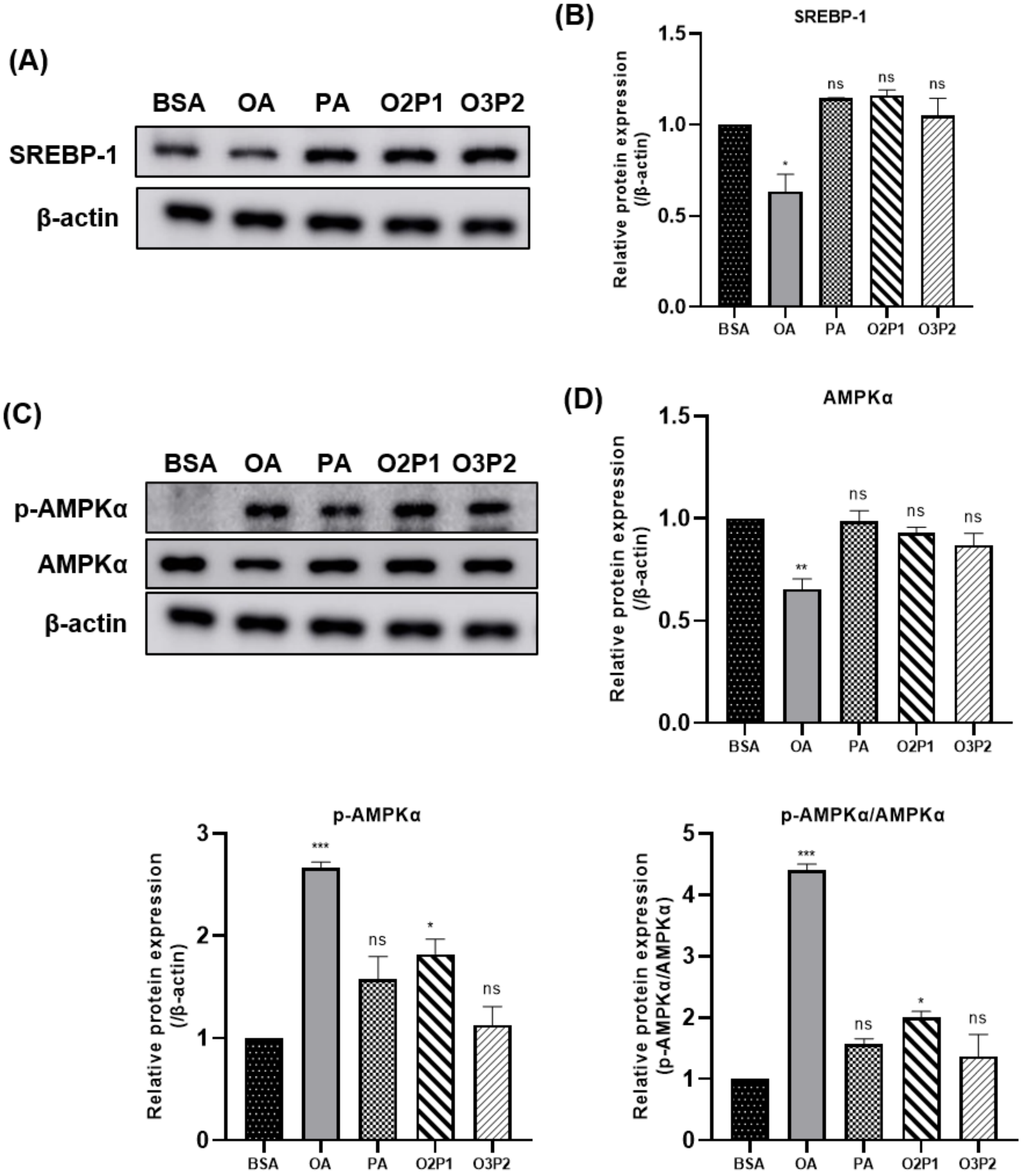

Figure S4. Selected lipogenic and AMPK-associated signaling responses to OA:PA treatment in SNU-449 cells. SNU-449 cells were treated for 24 h with BSA vehicle, OA (500 μM), PA (200 μM), O2P1, or O3P2 (500 μM total FFA). (A) Representative Western blot images of SREBP-1 and β-actin. (B) Densitometric quantification of SREBP-1 normalized to β-actin. (C) Representative Western blot images of phosphorylated AMPKα (p-AMPKα), total AMPKα, and β-actin. (D) Densitometric quantification of total AMPKα and p-AMPKα normalized to β-actin and the corresponding p-AMPKα/AMPKα ratio. Data are presented as mean ± SEM and expressed relative to the BSA control. *$p < 0.05$, **$p < 0.01$, ***$p < 0.001$ vs. BSA; ns, not significant.
